# MotifLeadDB: A Hierarchical Structural Data Set for Congeneric Ligand Binding Activity Change

**DOI:** 10.1021/acs.jcim.6c00128

**Published:** 2026-05-20

**Authors:** Nawoon Kim, Byunghyun Bae, Nuri Jung, Chaok Seok, Hahnbeom Park

**Affiliations:** † Biomedical Research Division, 58975 Korea Institute of Science and Technology, Seongbuk-gu 02792, Republic of Korea; ‡ Department of Metabiohealth, Institute for Cross-Disciplinary Studies, Sungkyunkwan University (SKKU), Suwon 16419, Republic of Korea; § Department of Chemistry, 200351 Seoul National University, Seoul 08826, Republic of Korea; ∥ Galux Inc., Seoul 08738, Republic of Korea

## Abstract

Predicting ligand binding free energy change by chemical substitutions is a critical task in drug optimization studies. Despite many efforts, existing computational methods show limited prediction accuracy, with one of the major reasons originating from poor training data. As a first step toward overcoming this challenge, we constructed MotifLeadDB, a structural model data set specially designed for the hit-to-lead optimization scenarios. For each receptor, known congeneric binder ligands were grouped by common scaffolds, and their complex structures were built with both ligand and side-chain optimization, so that the structural basis for the energy change upon functional group substitution could be directly inferred. To enhance the model confidence, ligand docking was guided by experimental complex structures sharing chemical similarity. The database consists of 342,489 protein–ligand complex structural models, which are grouped into 27,077 ligand scaffolds and are bound to 357 nonredundant receptors. The resulting structural models are also provided along with their confidence levels. We expect that the data set can be readily used for case studies to understand structure–activity relationships for target receptors of interest, as well as for training a computational model for estimating structure-based binding activity change.

## Graphical abstract

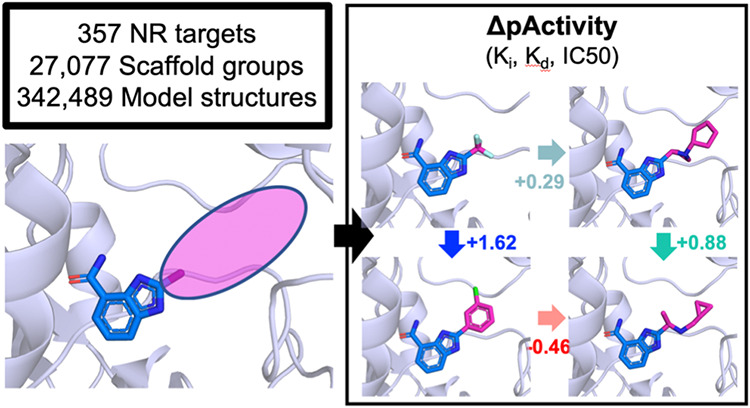

## Introduction

Optimizing protein–ligand binding affinity is one of central goals in structure-based drug design. However, computational prediction of absolute binding free energy (Δ*G*) remains challenging as it requires accurate estimation of thermodynamics ensembles.^1^ An alternative approach is to estimate relative binding free energy changes (ΔΔ*G*) between chemically related ligands such as congeneric ligands. Estimating ΔΔ*G* is valuable for two reasons: first, it is sufficient to guide hit-to-lead optimization scenarios (without absolute Δ*G*), and second, it is relatively more reliable and accurate than estimating Δ*G*, because many systematic errors cancel out within a shared receptor environment and the ligand scaffold.^1^ Therefore, many computational tools were developed to predict ΔΔ*G*,^2,3^ but machine-learning based methods were not very successful,^4^ and one of the major reasons was due to limited data.

In general, binding of a molecule to a receptor is experimentally measured in several ways, from IC_50_, most abundant data measuring activity, to *K*_d_ or *K*_i_, measuring binding equilibrium constant. Despite the difference between these measurements, it has been a common practice in many works^5,6^ to collect all these available activity-related data and deposit into a single data set in their raw or negative log values (p_Activity_) with measurement types. In this work, we define “ΔΔ*G*_app_,” as the pairwise difference in p_Activity_ values which refers to the activity change introduced by chemical modification.

Extraction of ΔΔ*G*_app_ from experimental data requires paired or grouped ligand–receptor structures that share a common binding site and ligand scaffold while differing in chemical substituents. However, such data are scarce. For example, chemical activity databases such as BindingDB,^5^ ChEMBL,^6^ or PubChem^7^ support extensive experimental binding or potency measurements, but lack structural models as their complexes with receptors, and require additional efforts to extract ΔΔ*G*_app_ across congeneric binders. Structural databases such as PDBbind^8^ or BioLIP^9^ supply experimentally determined complex structures, but the majority of those ligands lack binding modes for their congeneric variants. Recent model data sets include modeled complex structures that are built using template-based docking with similar known complexes,^10,11^ yet these are not well organized for the purpose of hit-to-lead scenarios, which is to obtain ΔΔ*G*_app_ across congeneric ligands.

To fill this gap, we constructed MotifLeadDB, a large-scale data set containing ΔΔ*G*_app_ values for congeneric ligand clusters together with their computed receptor-bound structural models. The data set was primarily generated by processing experimental BindingDB data: ligands corresponding to each protein target were grouped according to their common scaffolds, and structural models were built using template-based receptor-flexible docking. This procedure produced a hierarchically organized and easily accessible data set, providing structural context for activity changes arising from functional-group substitutions within congeneric ligand series.

## Methods

The overall data processing workflow is summarized in Figure 1a. The data set was constructed following 5 stages: (1) target selection, (2) ligand processing and scaffold grouping, (3) template complex preparation, (4) structural model construction, and (5) model confidence annotation. From the full set of BindingDB entries, data points were progressively filtered at each stage to ensure reliable structural foundations and minimize redundancy, reducing the number of modeled entries from the entire 3,147,844 entries in BindingDB to 342,489. Details of each stage are described in the following sections.

**Figure 1:**
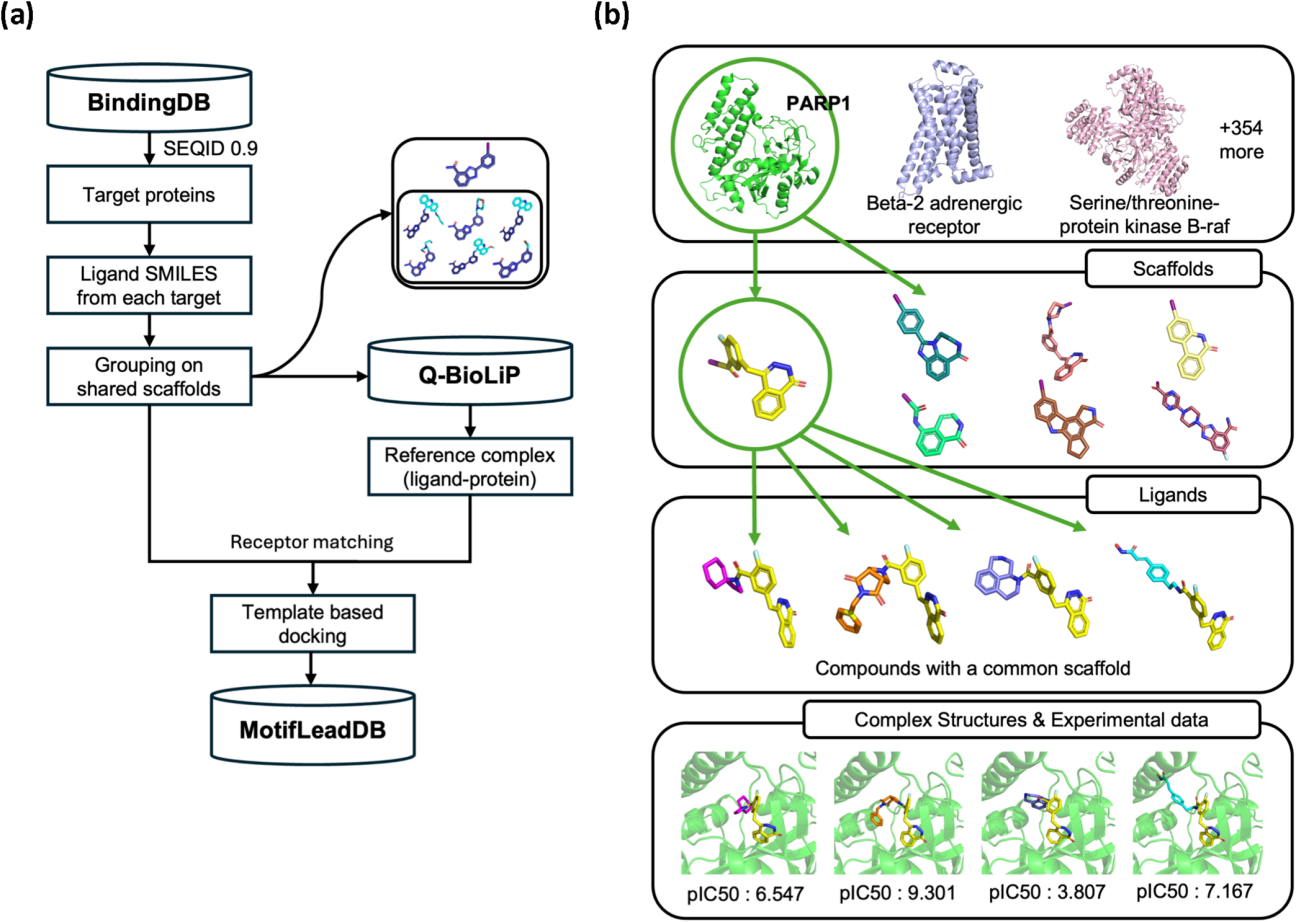
Overview of data processing workflow and hierarchical organization of MotifLeadDB. (a) Data processing workflow for generating structural models in MotifLeadDB. (b) Hierarchical organization of the resulting data set, consisting of protein targets, scaffold groups, ligands, and modeled receptor–ligand complexes linked with experimental activity data.

### Target Selection

Protein–ligand binding activity data were collected from the BindingDB database (April 2025 release) containing 3,147,844 entries. We first collected proteins with a single UniProt ID to ensure that each target corresponds to protein chains relevant for the ligand binding rather than the whole quaternary structure. Targets annotated with multiple UniProt identifiers, implying heteromultimeric receptor complexes, were excluded because of the ligand-binding site uncertainty. To ensure sufficient data for each target receptor, entries associated with fewer than 300 unique ligands with reported activity data were excluded. This threshold was chosen as a practical compromise between per-target ligand coverage and target diversity (Figure S1). Within each protein, the UniProt ID for the species (mostly human) with the largest number of ligands annotated was selected. To reduce sequence redundancy, the selected targets were clustered using MMseqs2 ^12^ easy-cluster (--min-seq-id 0.9 -c 0.8 --cov-mode 1). From each cluster, the target associated with the largest number of ligands was retained as the representative target in the final set. This process resulted in 756 receptor targets in total and their known binders.

In addition to our main strict target-selection pipeline, which was named as “Core set”, we additionally constructed a supplementary branch named “Diverse set” to broaden target-class coverage. All targets not included in the Core set yet containing more than 100 unique ligands were processed. Subsequent steps followed the same workflow as the main pipeline, except that scaffold groups containing fewer than two ligands were removed, to result in new 39 diverse targets (total 396).

In the following sections, we describe the pipeline to further curate the Core set.

### Ligand Processing and Scaffold Grouping

Ligands for the 756 selected receptors in BindingDB were extracted in their SMILES format with associated activity data. BindingDB entries with inequality signs on their activity records (e.g., “>”, “<”) were retained in our data set with signs. Records with construct-level variations such as mutant, fragment, and isoform categories, were removed from the Core set (kept only in the Diverse set). If multiple assay entries of the same measurement type existed for the same ligand–target pair, the median value was used as the representative activity value to reduce sensitivity to intersource variability and outliers, following suggestions from ChEMBL^6^ and Q-raKtion workflows.^13^ EC_50_ records were excluded, and the remaining activity values were converted to a negative logarithmic scale (p*K*_i_, pIC_50_, and p*K*_d_) for consistency across measurements. Downstream activity-difference calculations were performed only between ligands with the same measurement type. To focus on drug-like small molecules and reduce outliers from very large ligands, molecules with >35 heavy atoms were removed (retaining ∼82% of FDA-approved small-molecule drugs; Figure S2).

Ligands were allowed to have multiple scaffold definitions. Two different strategies were taken in parallel to annotate ligand scaffolds (Figure S3). An example of scaffold group construction with multiple ligands is illustrated in Figure S4a,b. The first strategy is to use BRICS fragmentation.^14^ For each ligand, the BreakBRICSbonds function in RDKit^15^ was applied, and all possible combinations of fragment pairs were generated, pairing one with a larger number of heavy atoms as the scaffold and the other as the substituent. Across all ligands binding to the same receptor, any group of ligands sharing their scaffold part were clustered together as a putative scaffold group. The second strategy is to apply maximum common substructure (MCS) analysis in RDKit. This strategy aims to detect scaffolds from fragments varying in their isosteres but are not breakable by the BRICS rule. Ligands were first clustered into a putative scaffold group having Tanimoto similarity (using the default RDKit fingerprint) above to a template ligand (generally closest ligand bound to the same receptor; see below for the template selection criteria). Then, MCS was annotated as the scaffold and the rest as substituents. Among 141,103 MCS scaffolds initially generated, 10,863 showing exact SMILES string match to BRICS scaffolds were discarded to reduce redundancy, leaving 130,240 unique MCS scaffolds.

The putative scaffold groups built above were then further filtered according to the following criteria: groups with fewer than five ligands or all ligands sharing identical activity values were excluded because of less information contained in the group. Ligands with substituents larger than 15 heavy atoms were removed as the portion of substituents became too large compared to their scaffolds (substituent statistics in Figure S15). For MCS-derived scaffolds, because more than two substituents can exist, molecules with the sum of substituent heavy atoms exceeding 15 heavy atoms were removed.

After ligand processing and scaffold grouping, receptors that did not retain at least one valid scaffold group were excluded, reducing the number of targets from 756 to 747.

### Template Complex Preparation

In the third step, experimental complex structures were selected for the template-based ligand docking. Template complex structures were retrieved from Q-BioLiP.^16^ Q-BioLiP was selected because it represents ligand–protein interactions based on quaternary structures rather than single-chain tertiary structures, providing more complete binding-site context for receptors whose ligands may interact with multiple chains. Here, template structures for a target were collected with conditions: the same UniProt ID, experimental structure with resolution ≤3 Å, and possessing at least one biological ligand molecule (as defined in Q-BioLiP). Any receptors which lack template complexes were removed. If more than a single template structure existed for a receptor, all of them were retained at this stage. To improve template–target consistency, template structures were further examined using SIFTS-based mapping.^17^ To reduce construct mismatch, templates containing mutations or other constructs within 5 Å of the bound ligand were distinguished from mutation-free templates. In the Core set, those entries having mutation-free templates were only collected. In the Diverse data set, if no suitable mutation-free template remained, mutant templates were retained with explicit annotations. Detailed criteria and template-level statistics are provided in Supporting Table S1.

Ions and cofactors in template complex structures were retained based on their biological importance (list of retained cofactors reported in Supporting Table S2). Templates were removed from the list if the ligand pose was affected by crystallographic contacts, which was defined as having at least three heavy atom contacts (<5 Å) between ligand and any other symmetric copies. Receptors lacking any remaining template complexes after these filtering steps were removed, reducing the number of receptors from 747 to 461.

For each template structure, we defined *modeling domains* that are sufficient and minimal for modeling ligand binding. All the receptor structures in our MotifLeadDB were provided with their modeling domains rather than their full structures in PDB.^18^ To get these, starting from original template structures in PDB, the receptor region surrounding each ligand was first cropped to include residues within 8 Å of any ligand atom, then the longest contiguous residue segment containing all these residues was selected. If a cofactor or ion was present in the selected domain, the domain was extended to also cover residues within an additional 4 Å to the cofactor or ion.

After defining the modeling domains, template complexes were systematically assigned to ligand scaffold groups based on the availability and similarity of experimental structures. Scaffold groups sharing one or more common ligands were first merged into a supergroup to ensure consistent receptor conformation (Figure S4c). For each supergroup, a single experimental complex was then selected as the template. If any ligand within the supergroup had an available experimental complex, that structure was directly used. When multiple experimental complexes were available, the one whose cocrystallized ligand showed the highest mean Tanimoto similarity to all scaffolds within the supergroup was selected. When no experimental complex was available, the search was extended to all template complexes of the same target, and the complex whose ligand exhibited the highest Tanimoto similarity to the scaffold was chosen. Templates with a maximum Tanimoto similarity <0.5 to any ligands were excluded from subsequent modeling. This threshold was chosen as a practical compromise to keep sufficient scaffold-template coverage; for instance, the fraction of retained ligand–template pairs drops from ∼25 to 14% when the similarity cutoff is adjusted from 0.5 to 0.6 (Figure S5). Upon merging, 50,555 ligands from 22,606 scaffolds with a Tanimoto similarity <0.5 to the newly defined templates were excluded. The distribution of average Tanimoto similarity between each scaffold group and its corresponding template within the supergroup is shown in Figure S5c, showing reasonable similarity above 0.5 to their templates for all the scaffolds included in the database.

The selected receptor structure was used for docking all scaffold groups belonging to the supergroup. This ensured consistent receptor geometry across related scaffolds while allowing scaffold-specific docking poses. To verify that the scaffold grouping and template assignment strategy maintained structural consistency, we examined ligands that appeared in multiple scaffold groups (Figure S6). Most ligands were linked to one to three scaffold definitions, and the overall alignment consistency across corresponding models was good (mean GDT-HA_lig_ = 0.60, see below “Model confidence annotation” for the definition), indicating that scaffold overlap generally produced comparable binding geometries.

### Structural Model Construction

In this step, ligands were docked into selected template structures at their modeling domains. Ligand docking started by building a representative ligand pose for each scaffold group. To do this, the group scaffold was first aligned to the selected template complex using g-align.^19^ Using this aligned scaffold as a positional reference, the shortest ligand in the group was subsequently aligned and used as the input for GALD (GALigandDock) docking.^20^ GALD was run in dockflex mode with two refinement iterations (Stage repeats = 2), maintaining a pool of 10 candidate poses at each stage (*n*_pool_ = 10) and initializing the protocol with five identical starting poses (initial_pool = 5). The procedure generated 10 final models per ligand (*n*_struct_ = 10) under local receptor side-chain flexibility and without positional restraints. When the resulting docking pose failed to reproduce the initial scaffold alignment (GDT-HA_lig_ < 0.7, see below “Model confidence annotation” for the definition), the receptor structure was prerelaxed using Rosetta FastRelax^21^ to better accommodate the ligand, and docking was repeated with the relaxed receptor. Once the representative complex of the shortest ligand was successfully modeled, it served as the reference structure for the remaining ligands in the group. These ligands were then aligned to the representative complex and docked with GALD following the same procedure, with Rosetta relax applied when the deviation against scaffold occurred.

After modeling all ligands in each scaffold group, complexes exhibiting excessive geometric distortion were filtered out using the Rosetta cart_bonded energy term (cart_bonded >10). Scaffold groups yielding fewer than five successfully modeled ligands were also excluded to ensure robustness for downstream ΔΔ*G*_app_ analysis. After these filtering steps, a total of 357 targets remained in the final data set.

### Model Confidence Annotation

The MotifLeadDB provides model structures by their confidence levels. Docked complexes were first filtered based on docking scores; those with positive interaction scores were excluded, as they indicate unfavorable conformations upon complex formation. Then models were classified into three confidence levels based on how well the modeled ligand preserves the scaffold position observed in the template. A ligand-version global distance test high-accuracy score (GDT-HA_lig_), originally developed as a protein structure accuracy metric,^22^ was computed for the scaffold portion of the modeled ligand relative to the aligned scaffold reference. For each of the four distance thresholds (0.5, 1.0, 2.0, and 4.0 Å), the fraction of corresponding scaffold atoms within the threshold was calculated, and GDT-HA_lig_ was defined as the mean of these four fractions; a value of 1.0 indicating perfect alignment. In the data set, complexes with GDT-HA_lig_ ≥ 0.7 were assigned to level 1 (most confident), those with 0.5 ≤ GDT-HA_lig_ < 0.7 to level 2, and the remainder to level 3.

We then applied an additional filtering based on the scaffold pharmacophore conservation. Pharmacophores were detected from complex structures by using OpenPharmaco.^23^ Hydrogen bond donors, acceptors, and aromatic ring centroids were considered as pharmacophore classes. A pharmacophore match was defined between a ligand atom and a template atom when they belonged to the same pharmacophore class and preserved atomic geometry. Position deviation had to be less than 1.5 Å, with an additional constraint for aromatic classes requiring the angle between ring normal vectors to be less than 30°. Then complexes with pharmacophore match ratio <0.80 were downgraded by one confidence level.

### Data Set Subset Definitions

To support analyses under different stringency levels and broader data set usage, we organized MotifLeadDB into five subsets (Table 1). The Diverse set is the broadest collection and combines entries from the original data set construction pipeline with entries from additional modeled targets incorporated to expand target-class coverage. The remaining subsets form a stricter Core branch derived from the mainstream target-selection pipeline. Within this branch, the Core set restricts entries to mutation-free templates, Core-NR further removes duplicated ligands with different scaffold definitions based on “primary scaffold assignments”, Core-NR-Act further restricts to entries with the same activity type with meaningful scaffold-level p_Activity_ variations (>0.2), and HC-Core further restricts the data to confidence level 1 entries. Here, the primary scaffold used from the Core-NR set was decided using a sequential priority order, listed from high to low priority: (1) the confidence level, (2) GDT-HA_lig_, (3) pharmacophore match ratio, and (4) more favorable Δ*H*.

**Table 1:** Definitions of MotifLeadDB Subsets and Their Definitions

	mutation-free template	nonredundant ligands	activity-type specific^a^	confidence level 1 only	num. targets	num. entries
Diverse	X	X	X	X	396	378,918
Core	O	X	X	X	357	342,489
Core-NR	O	O	X	X	357	97,173
Core-NR-Act	O	O	O	X	357	93,995
HC-Core	O	O	O	O	342	61,223

a Same measurement type within a scaffold with min-max p_Activity_ difference at least 0.2.

### MotifLead-Base, a Baseline ΔΔ*G*_app_ Prediction Model

To provide a simplistic baseline for future model developments using MotifLeadDB, we constructed a deep-learning model, named MotifLead-Base, that leverages the MotifLeadDB Core set as training data. The model first takes four atom-level features and one bond-level feature from a complex structure. Atom-level features are (i) SYBYL atom type, (ii) amino acid type (UNK for ligand), (iii) SASA measured by Shrake-Rupley algorithm,^24^ and (iv) partial charge brought from Rosetta^25^ for amino acids and MMFF94 ^26,27^ for ligands. Bond-order is used for the single bond-level feature. These features were processed through four layers of EGNN,^28^ an equivariant graph neural network, and converted to p_Activity_ value through a linear layer.

Data were split based on receptor sequence similarity. Target sequences were clustered with an identity threshold of 0.5. Targets in a cluster were grouped into one of training, validation, or test set, so that no target across data splits possess sequence identity higher than 0.5. In the data sampler, each scaffold group was first selected, then two ligands belonging to the scaffold group was chosen, putting higher sampling weights on those pairs with larger absolute ΔΔ*G*_app_

$$P_{\mathrm{ij}}=\exp\left(|{\Delta \Delta G}_{\mathrm{app},\mathrm{ij}}|\right)/Z;Z=\sum_{i,j}\exp\left(|{\Delta \Delta G}_{\mathrm{app},\mathrm{ij}}|\right)$$

Two losses were used with relative weights of 0.3 and 1.0: a MSEloss in p_Activity_ prediction and a MSEloss in their difference, ΔΔ*G*_app_. between selected pairs. The model was trained with Adam optimizer^29^ with a learning rate of 1.0 × 10^–4^. Dropout was set to 0.2. The training and inference codes, feature extraction code, model parameters, and data splits are freely accessible at https://github.com/HParklab/MotifLead.lite.

## Results

### Data Set Construction and Data Structure

The overall organization of MotifLeadDB is illustrated in Figure 1b. The following analysis is based on the Core set unless specified. The data set is structured hierarchically, beginning with 357 curated protein targets, grouped into 27,077 ligand scaffolds that encompass a total of 342,489 ligand–target pairs. Each scaffold connects multiple ligands sharing a common scaffold. This hierarchical design enables focused analysis of how small chemical substitutions within a shared scaffold influence binding complex structures and, ultimately, their potential energetic consequences. MotifLeadDB differs from other publicly available data sets in its “scaffold-centric” structure, size, and modeling technique employed; a summarized comparison is reported in Table 2 and Figure 2.

**Table 2:** Comparison of MotifLeadDB with Existing Protein–Ligand Structural Data Sets

	binding MOAD^30^	BindingNet^11^	MotifLeadDB
num. target proteins	11,058	802	357
redundant receptors	yes	no	no
num. ligands	20,387	65,918	101,151
grouped by ligand scaffold	no	no	Yes
			27,077
complex structures provided	41,409	69,816	342,489

**Figure 2:**
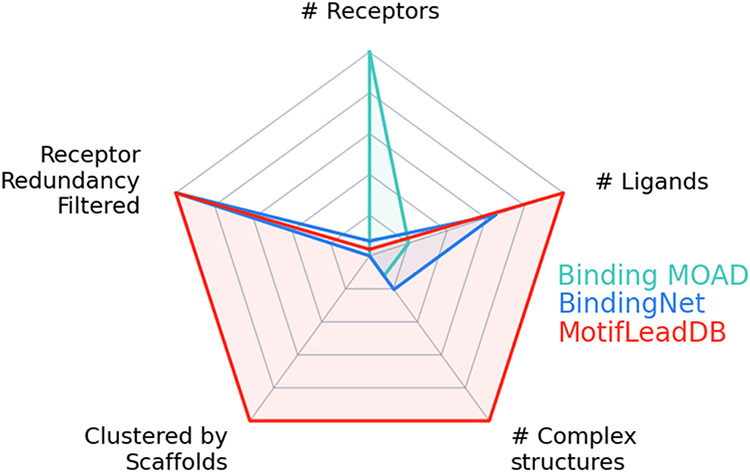
Visual comparison of MotifLeadDB and existing protein–ligand structural data sets. Numerical features (number of targets, ligands, and complex structures) are normalized within each category for visualization, whereas binary features (clustered by scaffolds and receptor redundancy filtered) are encoded as 0 (no) or 1 (yes).

### Target Distribution and Data Set Statistics

#### Data Set Partitioning and Subset Statistics

After filtering through the full pipeline, the curated Core set comprised 342,489 protein–ligand complex structures. By incorporating supplementary targets to broaden target-class coverage and more tolerable criteria on mutations and construct variants, we further added entries, yielding the Diverse set with a total of 378,918 entries (Figure 3a). From the original curated collection, we defined the Core set as the principal subset for downstream analyses. Additional subsets derived from the Core branch included Core-NR (97,173), Core-NR-Act (93,995), and HC-Core (61,223).

**Figure 3:**
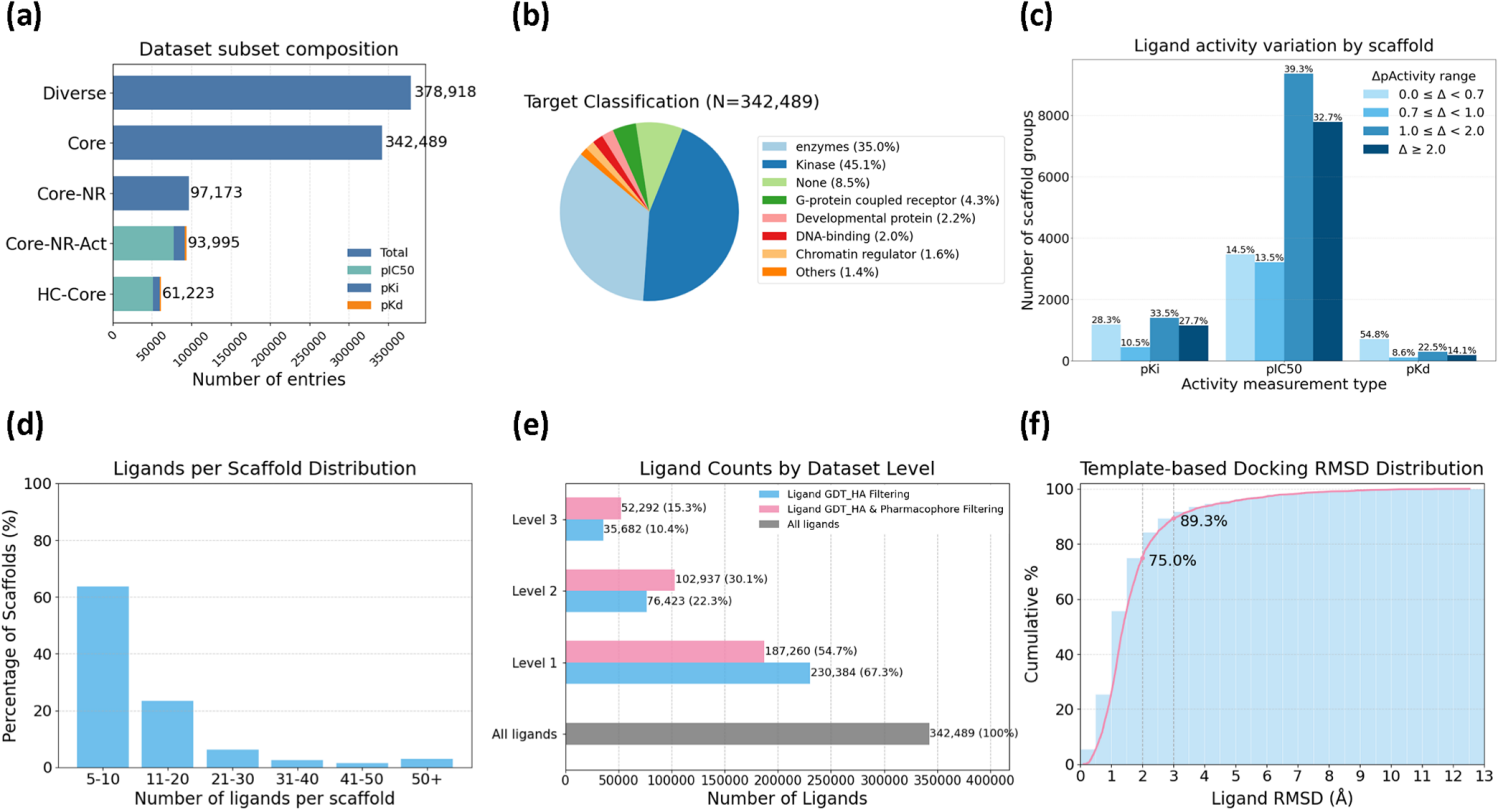
Data set composition and statistics of MotifLeadDB. (a) Composition of the MotifLeadDB subsets and their entry counts across the curated, Core, Core-NR, Core-NR-Act, HC-Core, and Diverse sets. (b) Target distribution across functional categories, including kinases (45.1%), other enzymes (35.0%), GPCRs (4.3%), and smaller fractions of other protein classes. (c) Distribution of maximum difference in activity values per scaffold group, shown for each activity measurement type. About 30% of scaffold groups showed a range of at least 2.0 (i.e., >100 fold change). (d) Distribution of the number of ligands per scaffold group. (e) Distribution of ligand confidence levels based on scaffold alignment similarity (GDT-HA_lig_) and pharmacophore conservation (definitions in the main text). (f) Benchmarking the structural accuracy of our modeling pipeline on known complexes. Cumulative distribution of ligand RMSD values to their crystal structures are shown. 75.0 and 89.3% of docked poses are within 2 and 3 Å, respectively.

#### Target Distribution

The curated data set comprises a total of 342,489 ligand–target pairs after filtering following the procedure reported in Figure 1 and Methods. Targets were broadly distributed across functional categories (Figure 3b), with kinases (45.1%) and enzymes (35.0%) constituting the majority, followed by GPCRs (4.3%) and smaller fractions of DNA-binding proteins, chromatin regulators and other classes. Entries labeled as “None” correspond to targets lacking specific functional annotation in UniProt, while “Others” represent proteins not belonging to the major categories listed above. This distribution in receptor types reflects the tendency in current ligand activity studies, even after 90% sequence clustering, which have traditionally focused on kinases, enzymes, and GPCRs. To address the lack of diversity in the set, supplementary targets were introduced to the Diverse subset in addition to original targets to broaden target-class coverage (target-class distribution of these added targets shown in Figure S7).

#### Activity Distribution

In the Core set, retained activity-type annotations were dominated by IC_50_ (87.5%), followed by *K*_i_ (10.5%) and *K*_d_ (2.0%), whereas EC_50_ records were excluded during activity processing (Figure S8a). Downstream comparisons were performed only among ligands sharing the same activity type within each scaffold group. We additionally evaluated provenance consistency across all same-type scaffold groups in the Core set and found that 72.2% were classified as single-source, 27.1% as mixed-source, and 0.7% as not evaluable (Figure S8b). Among scaffold groups with mixed sources, 46.3% of pairs exhibited Δp_Activity_ <0.7, suggesting potential limitations due to experimental uncertainty that may obscure modelable signal. Approximately 30% of scaffold groups showed a within-group activity range of at least 2.0 p_Activity_ units between the highest and lowest retained values (i.e., >100-fold difference), indicating that ligands sharing the same scaffold often exhibit substantial activity differences (Figure 3c). To assess whether these large ranges could reflect cross-source aggregation, we further examined the range-defining max–min ligand pairs for these groups using PMID-, DOI-, and patent-based source clustering (Figure S8c). Among scaffold groups with Δp_Activity_ ≥ 2.0, 71.7% of the range-defining pairs were linked to a single source cluster, whereas 27.6% were associated with distinct source clusters (0.8% not evaluable). Together, these results suggest that MotifLeadDB contains substantial within-scaffold activity variation while preserving substantial source consistency.

#### Scaffold Group Statistics

Scaffold-based grouping revealed that the majority of scaffold groups contained 5–10 ligands, whereas progressively fewer groups included more than 20 ligands (Figure 3d). More analyses on scaffold group characteristics are reported in Figure S9. The number of heavy atoms in unique scaffolds were distributed as a bell-shape with a median value of approximately 22 (Figure S9a). The top 10% of scaffold types accounted for around 39% of all ligands, demonstrating the dominance of a limited number of recurrent scaffolds (Figure S9b). Representative examples of the top 20 most frequent scaffolds are shown in Figure S9c.

We additionally summarized similar statistics for substituents (Figure S10). Distribution in the number of heavy atoms in unique substituents (Figure S10a) showed a broad range with a median around 8–14 heavy atoms. Counting their occurrences (Figure S10c), small functional groups such as methyl, halogens, and hydroxyl units appeared most frequently, reflecting isostere-level substitutions rather than large fragment insertions. The number of distinct substituents per ligand was typically one or two (Figure S10b), indicating that ligands within each scaffold group differ mainly by a small number of peripheral substitutions rather than extensive structural changes.

### Confidence of Structural Models

#### Confidence Annotation Statistics

Ligand–template alignments were classified into three confidence levels reflecting both structural and pharmacophore similarity to their experimental templates (see Methods). After applying this combined criterion, 187,260 ligands (54.7%) were assigned to Level 1 (most confident), 102,937 (30.1%) to Level 2, and 52,292 (15.3%) to Level 3 (least confident, Figure 3e). The GDT-HA_lig_ distribution (Figure S11a) indicated that over 60% of ligands achieved GDT-HA_lig_ ≥ 0.7, confirming that most modeled ligands closely follow their template scaffolds. Pharmacophore conservation scores showed a similar trend (Figure S11b), with the majority of ligands maintaining ≥0.8 feature similarity. Together, these results demonstrate that the final confidence levels effectively integrate both geometric and chemical agreement, supporting the reliability of the modeled complex data set.

#### Structural Robustness of Models

To assess the structural reliability of our modeled complexes, we examined cases where the modeled ligands actually have their corresponding crystallographic structures. A total of 2425 instances were benchmarked. RMSD analysis showed that 75.0% of ligands achieved RMSD ≤2 Å relative to the crystallographic pose, and 89.3% were within 3 Å (Figure 2f). Further analysis showed that RMSD tended to increase with decreasing model confidence (Figure S12a), with 77.2% of Level 1 ligands having RMSD ≤2 Å. In addition, structural deviations were mainly observed in peripheral substituent regions, whereas the core scaffold geometry was largely preserved (Figure S12b). It should be noticed that this benchmark was done in favorable evaluation scenarios when close templates exist (Tanimoto similarity >0.5). With more generic docking scenarios, such as template-based docking with no similarity specification or a blind docking, success rates are reported to be much lower: 42 and 22%, respectively, when tested on PDBbind set.^8^

Because this benchmark subset was defined by reference-structure availability rather than actual data set distribution, we compared its target-class and confidence-tier distributions with those of the parent Core set to assess representativeness (Figure S12c). The benchmark subset broadly preserved the dominant kinase/enzyme composition of the Core set, but underrepresented GPCR and receptor classes, and was enriched for higher-confidence entries, particularly Level 1 models. Accordingly, the reported RMSD accuracy should be interpreted in the context of this benchmark composition.

### Flexible Side-Chain Effects on Binding Geometry

Unlike other structural model databases, MotifLeadDB permits side-chain modifications in deposited models following ligand fragment substitutions. The degree of change is sometimes beyond a local minimization level, because the side-chain modeling by GALD allows rotameric change as well. This rather aggressive modeling – compared to previous works – can be first justified by the benchmark results of GALD for receptor-flexible docking with side-chain remodeling, which improved the ligand pose accuracy by 5%.^20^ Here, we qualitatively investigated if this known advantage of side-chain modeling transfers to our constructed data set.

In principle, adjustments in side-chain conformations can improve structural complementarity between ligands and binding pockets. We examined pockets containing residues with significant side-chain remodeling (any Δχ ≥ 60°). Among these flexible-side-chain cases, 67.2% of the pockets showed a decrease in clashing residues (Figure 4a), indicating that side-chain reorientation effectively alleviated steric conflicts. Furthermore, new intermolecular interactions occurred in many cases, suggesting that side-chain flexibility contributes to more realistic binding geometries. Representative examples of these adaptive changes are described in the following paragraphs in detail (Figure 4b,c).

**Figure 4:**
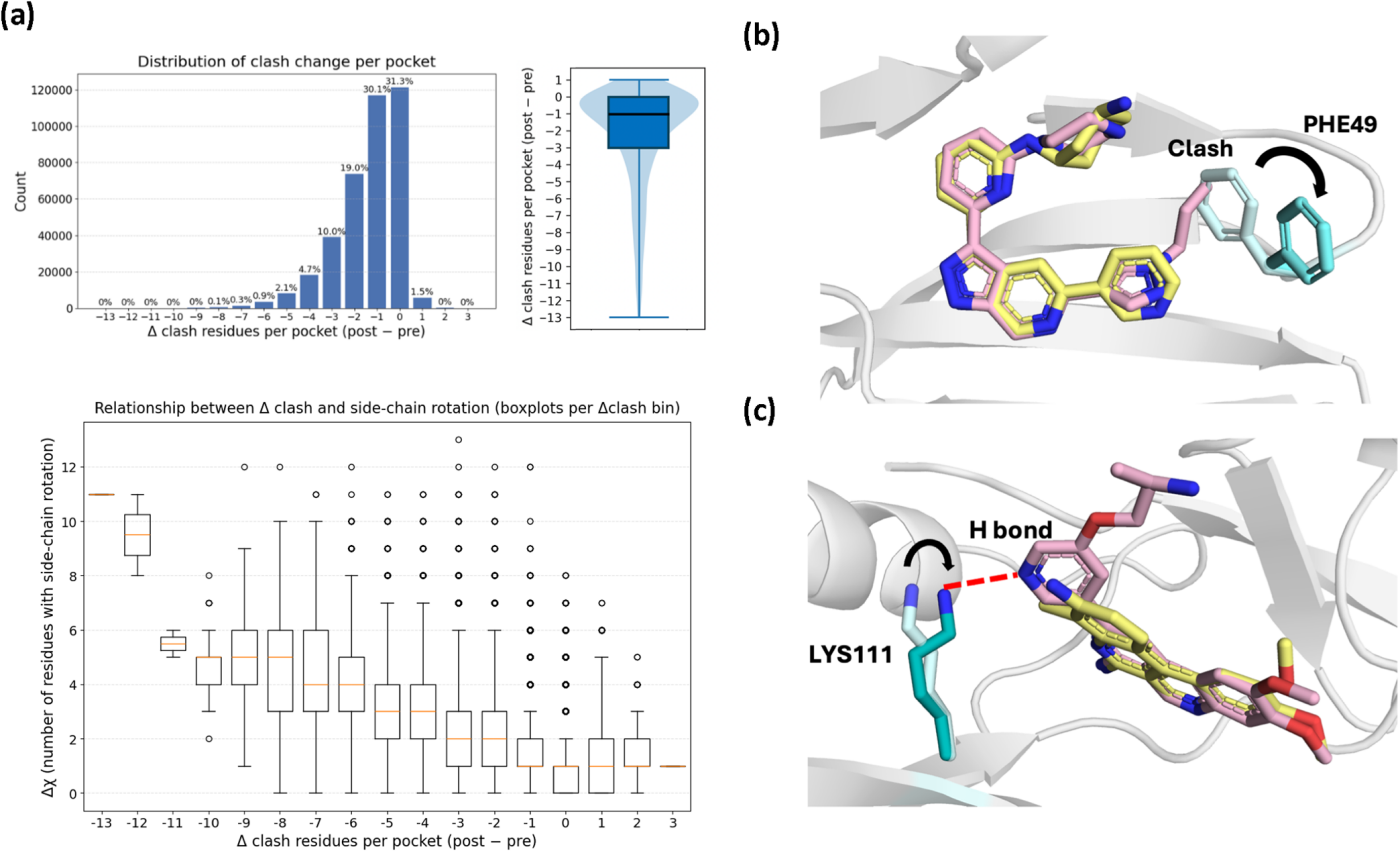
Representative examples of side-chain flexibility effects on binding geometry. Ligands/protein residues from the template complex are shown in yellow/light-cyan, and those from the new ligand docking results are shown in pink/turquoise. (a) Distribution of the change in clashing residues per pocket (Δ clash residues, post–pre). (b) Reduction of steric clash by outward rotation of Phe49. (c) Formation of a new hydrogen bond interaction between Lys111 and ligand.

To further characterize the overall behavior of side-chain remodeling, we analyzed the statistical distribution of χ-angle changes across all modeled complexes (Figure S13). χ_1_ and χ_2_ angles were considered. Among pockets containing at least one residue with a large side-chain rotation (Δχ ≥ 60°), approximately 60% exhibited two or fewer such changes, indicating that major conformational adjustments were generally limited and localized rather than widespread (Figure S13a). The residues showing frequent remodeling were predominantly polar residues such as His and Asp (Figure S13b). A mild correlation was observed between the number of side-chain rotations and the reduction in the number of clashing residues, suggesting that local side-chain flexibility contributes to alleviating steric conflicts (Figure S13c). Furthermore, interaction-type analysis showed that major noncovalent interaction types displayed a balanced distribution of increases and decreases, resulting in negligible net changes overall (Figure S14). This overall result suggests the major outcome of side-chain changes is clash-relieving, with a minor effect to form additional favorable interactions while relieving clashes. This is further explained by the following case studies.

#### Case 1. Reduction of Steric Clash (Figure 4b)

This case illustrates how local side-chain flexibility enables the binding pocket to adapt to a bulkier ligand. In the template complex (PDB: 5DIA; template ligand shown in yellow), Phe49 adopts an inward-facing conformation that accommodates the original ligand without steric conflict. When the ligand is replaced with a larger analog (pink), direct superposition leads to a steric clash with Phe49. In the modeled complex, however, Phe49 rotates outward, resolving the clash and creating sufficient space for the new substituent while preserving the key interactions present in the template structure. The two ligands also differ in their experimentally measured affinities: the template ligand has p*K*_i_ = 9.40, whereas the replacement ligand shows p*K*_i_ = 10.41. This +1.01 increase in p*K*_i_ (≈10-fold improvement) is consistent with the improved vdW interactions in the bulkier ligand after resolving the superposition-induced steric clash.

#### Case 2. Formation of new interactions (Figure 4c)

This case demonstrates how ligand-induced side-chain flexibility can create new stabilizing interactions within the binding pocket. In the template complex of 2R7B (template ligand shown in yellow), the side chain of Lys111 is oriented away from the ligand and does not participate in any polar contacts. The original ligand occupies a smaller region of the pocket, leaving Lys111 too distant to form a hydrogen bond. When the ligand is replaced with a bulkier analog (shown in pink), the extended substituent reaches toward the Lys111 region. In response, Lys111 rotates inward and moves deeper into the pocket, enabling the formation of a new Lys111–ligand hydrogen bond that is absent in the template complex. This additional interaction improves local complementarity and stabilizes the modeled binding pose. Experimentally, the template ligand exhibits IC_50_ = 60 nM (pIC_50_ = 7.22), whereas the replacement ligand shows pIC_50_ = 8.52, corresponding to an increase of ΔpIC_50_ = +1.30 (≈20-fold higher potency). The emergence of the new hydrogen bond is consistent with this experimentally observed improvement in potency.

### Data Set Applications for SAR Study

To illustrate how MotifLeadDB can support structure-guided SAR interpretation under more controlled assay conditions, we examined a representative congeneric series from the Core set, restricting the analysis to ligands sharing the same measurement type and the same literature source (Figure 5).

**Figure 5:**
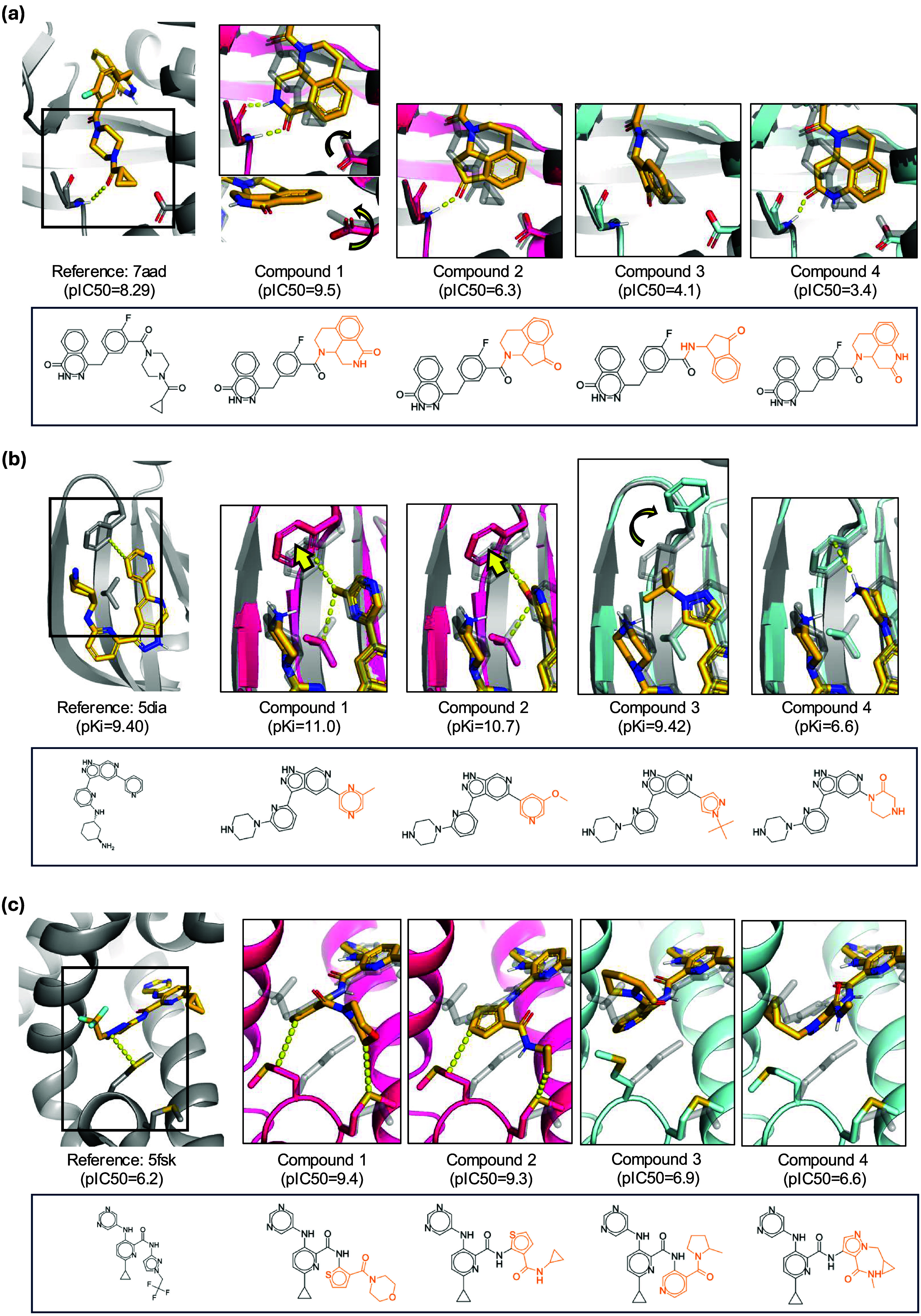
Representative structure-based rationales for SAR trends across three ligand series using MotifLeadDB models (a–c). For each case, the left panel shows the reference experimental binding mode, and the right panels show modeled poses of representative analogs with their activity values. Variable parts are highlighted by black boxes.In the right panels, template structures are overlaid in transparent gray sticks along with structures for selected complexes for comparison.

#### Case 1 (Figure 5a)

Slight rotation of Asp770 allows repositioning of the ligand to avoid clash when the substituent is bulky. In Compound 1, this local pocket rearrangement, along with formation of two hydrogen bonds with Arg878, provides a plausible structural rationale for its strongest activity in this series (pIC_50_ = 9.5). Compounds 2–4 do not maintain the same dual hydrogen-bonding pattern and therefore adopt less favorable interaction geometries than Compound 1.

#### Case 2 (Figure 5b)

Introduction of a small methyl group (Compound 1) or a methoxy group (Compound 2) could accommodate improved vdW packing with slight rotation of Val52 and Phe49 side-chains. This ligand-induced receptor change and improved packing is consistent with the experimental activity improvement with respect to the reference experimental structure. The Compound 3 has a bulkier group compared to former ligands, and binding will be available only with Phe49 rotation to reduce clash. This structural model explains why Compound 3 has comparable activity to the reference but also worse than Compound 1 or 2. The Compound 4 (p*K*_i_ = 6.6) showed substantially reduced activity, which can be explained by the positively charged group facing Phe49.

#### Case 3 (Figure 5c)

Met713 reorients outward, altering the local pocket geometry. In Compounds 1 (pIC_50_ = 9.4) and 2 (pIC_50_ = 9.3), this rearrangement is accompanied by additional vdW interactions in the Met713/Met714 region relative to the reference structure, consistent with their improved activity. In contrast, Compounds 3 (pIC_50_ = 6.9) and 4 (pIC_50_ = 6.6) do not reproduce these additional interactions, providing a plausible structural rationale for their lower activity.

Taken together, this series highlights multiple SAR patterns captured by the modeled complexes: strong activity gains can arise when modest substituent growth is accommodated by local side-chain remodeling and favorable hydrophobic packing, whereas other modifications may yield structurally less favorable outcomes and result in either little net benefit or substantial activity loss, depending on how well the remodeled pocket accommodates the new substituent.

### Data Set Applications for ΔΔ*G*_app_ Prediction Model Development

To facilitate the model development using MotifLeadDB, we have built a baseline deep neural network, MotifLead-Base, trained on a 80% subset of the MotifLeadDB Core set. The model exhibits a mean ΔΔ*G*_app_ error of 0.8, when tested on separate data containing 1820 ligands from 95 unique scaffold groups (full data available in https://github.com/HParklab/MotifLead.lite). This level of error is comparable to the mean standard deviation of experimental p_Activity_ values measured across intragroup ligands, indicating that the model’s performance is effectively close to random. This result highlights further model development and data curation is necessary for more robust ΔΔ*G*_app_ prediction. We believe the baseline model, along with the provided data set, should serve as a starting point for further model development efforts.

## Discussion

MotifLeadDB is a large-scale structural data set for the study of generic hit-to-lead optimization scenarios, containing a total 101,151 unique ligands with experimental activity values associated with their structural models. The database is designed to enable systematic analysis and to provide structural bases for the scaffold-level variations in ligand binding activity. Importantly, this scaffold-based organization supports ΔΔ*G*_app_ estimation directly originating from chemical diversity within a shared scaffold correspondence. Moreover, incorporating flexible side-chain docking enabled realistic local rearrangements of binding-site residues, producing more plausible binding geometries. This flexibility helped alleviate steric conflicts and restore missing nonbonded interactions, thereby enhancing structural complementarity within the binding pocket. Finally, the data set is provided with per-structure confidence estimates, which allows users to select and customize the data set usage on their own purpose.

Despite these advantages, several limitations remain. Explicit solvent molecules were not included during docking, and therefore water-mediated or long-range electrostatic effects to ΔΔ*G*_app_ are not represented accurately. In addition, all complexes were generated computationally, and docking-based procedures inherently involve a degree of structural uncertainty. Inherent variability in experimental data presents an additional concern; for example, activity measurements obtained under diverse assay conditions may introduce noise into quantitative analyses. Construct mismatch can be also a potential limitation. Particularly for kinases and GPCRs, a Uniprot ID based template selection can often ignore any mutations in proteins which can cause orders-of-magnitude errors in the bioactivity data.^31^ To minimize this concern, we prioritized wild-type templates as much as possible, and removed all the BindingDB entries containing any construct mismatches from our Core set. However, annotation errors that our automated pipeline could not capture may still exist, and careful inspection on usage is recommended.

Proper grouping of congeneric ligand series is an important step toward constructing a data set such as MotifLeadDB. In this work, a combined approach of BRICS rule and Maximum Common Substructure (MCS) was taken to this end. Alternative approaches like Matched Molecular Pair Analyses (MMPA)^32^ can be considered as well. As pointed out by the authors, MMPA approach is conceptually similar to MCS but computationally more efficient. We believe our strategy of using MCS was reasonable within our pipeline, because the number of compounds subject to clustering was reasonably small (maximum 200) due to the fingerprint-based filtering prior to MCS. Yet, alternative grouping using MMPA and comparing against the current MotifLeadDB on their diversity will be worth investigating in the future.

In the current version, only monomeric proteins annotated with a single UniProt identifier were included to ensure unambiguous correspondence between amino acid sequences and modeled structures. Future extensions could incorporate multimeric receptor complexes and explicit solvent representations to further enhance the biological realism and general applicability of the data set. We freely open this data set to the public with two intended outcomes: enabling convenient structure–activity relationship studies for medicinal chemists and supporting the development of ligand binding activity prediction models for computational chemists.

## Supplementary Material



## Data Availability

The MotifLeadDB, including modeled receptor–ligand complex structures and associated metadata, is freely available for download at https://zenodo.org/records/19116065. The code and weights for *MotifLead-Base*, a ΔΔ*G*_app_ prediction model, can be accessed through the github repository via https://github.com/HParklab/MotifLead.lite. The G-align code and instructions can be found through the github repository via https://github.com/seoklab/galign.

## Notes

Detailed descriptions of the MotifLeadDB, including construct-consistency screening and template selection, types of ligands retained in the DB, organization and metadata of the DB are reported as supporting tables. Scaffold annotation scheme illustration, various statistics on selection thresholds over number of entry counts, various statistics of the DB on their unique scaffolds, substituents, confidence distribution, and side-chain remodeling results are reported in supporting figures (PDF)
