# Supplementary material for: MotifLeadDB: A Hierarchical Structural Data Set for Congeneric Ligand Binding Activity Change
Source: J Chem Inf Model. 2026 May 20;66(11):6376–86. doi: 10.1021/acs.jcim.6c00128 (PMC13250906; doi:10.1021/acs.jcim.6c00128)

Supplementary Information to

MotifLeadDB: A Hierarchical Structural Dataset for Congeneric Ligand Binding Activity Change

Nawoon Kim[1,2], Byunghyun Bae[1,3], Nuri Jung[3], Chaok Seok[3,4], Hahnbeom Park[1*]

[1] Biomedical Research Division, Korea Institute of Science and Technology, Republic of Korea 02792
[2] Department of Metabiohealth, Institute for Cross-disciplinary Studies, Sungkyunkwan University (SKKU), Suwon, Republic of Korea 16419
[3] Department of Chemistry, Seoul National University, Seoul, Republic of Korea
[4] Galux Inc., Republic of Korea 08826

* Correspondence to: hahnbeom@kist.re.kr

Table of Contents

Supplementary Table S1. Construct-consistency screening and template-selection summary.

Supplementary Table S2. List of ions and cofactors retained from Q-BioLiP template complexes.

Supplementary Table S3. Directory structure and file organization of the MotifLeadDB.

Supplementary Table S4.Data schema and file organization of MotifLeadDB.

Supplementary Table S5. Description of metadata columns in MotifLeadDB.

Supplementary Figure S1. Trade-off between minimum ligand-count threshold and retained targets.

Supplementary Figure S2. Distribution of heavy-atom counts for FDA-approved small-molecule drugs.

Supplementary Figure S3. Examples of scaffold annotation using BRICS and MCS.

Supplementary Figure S4. Scaffold group formation and supergroup unification.

Supplementary Figure S5. Tanimoto similarity statistics in template assignment.

Supplementary Figure S6. Distribution and structural consistency of ligands assigned to multiple scaffold groups.

Supplementary Figure S7. Supplementary targets added to expand the Diverse set.

Supplementary Figure S8. Activity-type composition and provenance-aware activity analysis in the Core set.

Supplementary Figure S9. Scaffold statistics.

Supplementary Figure S10. Substituent statistics.

Supplementary Figure S11. Confidence assessment statistics.

Supplementary Figure S12. Additional analyses of the crystal-validated Core benchmark subset.

Supplementary Figure S13. Statistical summary of side-chain remodeling across the dataset.

Supplementary Figure S14. Changes in noncovalent interactions upon side-chain remodeling.

Supplementary Figure S15. Distribution of substituent heavy-atom counts before applying the 15-heavy-atom cutoff.

Supplementary Table S1. Construct-consistency screening and template-selection summary. BindingDB target-name parsing, SIFTS-based pocket checks, and SIFTS-based whole-structure UniProt mapping are provided.

Core excludes entries flagged by target-name construct parsing and entries whose referenced templates show (i) pocket amino-acid mismatches to the target UniProt or (ii) pocket residues mapping to multiple UniProt accessions (≤5 Å; SIFTS-based).

(a) Entries were flagged using semi-structured BindingDB target-name annotations (mutant/fragment/isoform) and excluded from the Core dataset.

	Affected unique targets	Affected unique templates	Excluded entries
**Mutant target name**	2	10	4,205
**Fragment target name**	2	2	92
**Isoform target name**	4	26	14,176

(b) Template complexes were classified by SIFTS mapping and residue-level pocket checks (≤5 Å from the bound ligand) to screen construct mismatches.

	Affected unique templates	Affected unique targets	Excluded entries
**Pocket-clean (single UniProt chain)**	1,794	355	350,472
**Pocket-clean (multi-UniProt chain)**	37	20	15,502
**Pocket mutation**	46	25	6,928
**Pocket mixed-UniProt**	1	1	7

(c) Whole-structure construct features assessed by SIFTS-based UniProt mapping over the entire chain. Statistics are descriptive (no exclusion is applied based on these counts).

	Affected unique templates	Affected unique targets
**Fusion**	44	24
**UniProt coverage < 0.5**	803	142
**UniProt coverage < 0.7**	1,072	215
**UniProt coverage < 0.9**	1,396	287

Definitions:

Pocket-clean (single UniProt chain): pocket residues map to the target UniProt only and show no amino-acid mismatches to UniProt (≤5 Å from ligand), with a single UniProt assignment for the mapped chain.

Pocket-clean (multi-UniProt chain): the mapped chain may carry multiple UniProt assignments at the chain level, but pocket residues map exclusively to the target UniProt and show no amino-acid mismatches (≤5 Å).

Pocket mutation: at least one pocket residue (≤5 Å) mapped to the target UniProt shows an amino-acid mismatch between PDB and UniProt.

Pocket mixed-UniProt: pocket residues (≤5 Å) map to multiple UniProt accessions.

Fusion: target-UniProt-mapped chain also contains mapping to one or more additional UniProt accessions.

UniProt coverage: union of SIFTS-mapped UniProt residues divided by the full UniProt sequence length. As no universal threshold for "domain fragment" exists, counts at multiple cutoffs are reported.

Supplementary Table S2. List of ions and cofactors retained from Q-BioLiP template complexes.

Type of HET	Ligand ID
**Metal/Ion**	CU, FE, MG, NI, MN, K, NA, CO, ZN, CA, CL, CD
**Cofactor**	• Heme Related (HEM, HEC, HEO, HEA)<br>• Nucleotides/Nucleosides Related<br>• Plinder cofactors (Coenzyme A, Orthoquinone residues (LTQ, TTQ, CTQ), Glutathione, S-adenosylmethionine, Nicotinamide-adenine dinucleotide, Dipyrromethane, Thiamine diphosphate, Topaquinone, Tetrahydrofolic acid, Molybdopterin, Biopterin, Flavin Mononucleotide, Ubiquinone, Flavin adenine dinucleotide, Heme, Ascorbic acid, Adenosylcobalamin, Biotin, Coenzyme M, Factor F430, Lipoic acid, MIO, Pyridoxal 5'-phosphate, Menaquinone, Phosphopantetheine, Pyrroloquinoline Quinone, Coenzyme B)

Supplementary Table S3. Directory structure and file organization of the MotifLeadDB. MotifLeadDB is distributed with core.csv as the main strict dataset and diverse_only.csv as the additional set of entries not included in the Core dataset. Additional subset-specific tables derived from the Core dataset are provided in the tables directory. Modeled receptor–ligand complexes are stored separately for the Core and diverse-only branches and are organized by scaffold group. The metadata directory provides summary tables, column descriptions, and dataset-level statistics. The README file explains how the tables and modeled complexes are linked, enabling straightforward retrieval of the desired subset.

```
MotifLeadDB/
├── README.md                          # dataset overview and usage instructions
├── core.csv                           # main strict dataset (Core set)
├── diverse_only.csv                   # additional entries outside the Core set
├── tables/                            # derived subset tables from the Core set
│   ├── core_nr.csv                    # non-redundant Core subset
│   ├── core_nr_act.pKi.csv            # Core-NR-Act subset (pKi)
│   ├── core_nr_act.pIC50.csv          # Core-NR-Act subset (pIC50)
│   ├── core_nr_act.pKd.csv            # Core-NR-Act subset (pKd)
│   ├── high_core.pKi.csv              # high-confidence Core-NR-Act subset (pKi)
│   ├── high_core.pIC50.csv            # high-confidence Core-NR-Act subset (pIC50)
│   └── high_core.pKd.csv              # high-confidence Core-NR-Act subset (pKd)
├── core_structures/                   # modeled complexes corresponding to the Core branch
│   └── P49356_2F0Y_3MN_1/             # example scaffold-group folder
│       ├── P49356_2F0Y_3MN_1_0.pdb
│       ├── P49356_2F0Y_3MN_1_1.pdb
│       └── P49356_2F0Y_3MN_1_2.pdb
├── diverse_only_structures/           # modeled complexes corresponding to the diverse-only extension
│   └── Q99523_4N7E_2JQ_4/             # example scaffold-group folder
│       ├── Q99523_4N7E_2JQ_4_0.pdb
│       ├── Q99523_4N7E_2JQ_4_1.pdb
│       └── Q99523_4N7E_2JQ_4_2.pdb
└── metadata/                          # summary tables and annotations
    ├── MotifLeadDB_summary.csv        # overall dataset summary
    ├── MotifLeadDB_model_list.csv     # model-level index table
    ├── MotifLeadDB_ligand_list.csv    # ligand-level summary table
    ├── MotifLeadDB_target_list.csv    # target-level summary table
    ├── MotifLeadDB_bindingdb_list.csv # BindingDB mapping table
    └── partition_stats_summary.csv    # summary statistics for dataset partitions
```

Supplementary Table S4. Data schema and file organization of MotifLeadDB. This table summarizes the overall organization of MotifLeadDB, including the roles of the CSV tables.
(a) Model annotation table: identifiers and model-level metrics for each modeled protein–ligand complex (e.g., docking enthalpy terms, GDT-HA, pharmacophore score, confidence level).
(b) Ligand annotation table: ligand structures (SMILES) and experimental activity values aggregated from BindingDB, with cross-references to external databases (PubChem, ChEMBL, DrugBank, and ZINC, when available).
(c) Target metadata table: protein target metadata and UniProt-based annotations (e.g., UniProt ID, organism, functional annotation, sequence).
(d) BindingDB-derived annotation table: BindingDB identifiers and experimental context metadata associated with the activity measurements used in MotifLeadDB (e.g., pH and temperature, when available).

# Model annotations

model_ID	confidence_level	MotifLead_ligand_ID	scaffold_ID	group_SMILES	substituent_SMILES	ligand_SMILES	dH	gdt_ha	pharmacophore_score	pKi	pIC50	pKd	pEC50
Q9Y5Y4_6D27_FT4_9_1	1	Q9Y5Y4.MLD104703	Q9Y5Y4_6D27_FT4_9	[*]C1CCc2c(CC(=O)O)c3ccc(Cl)cc3n2C1	[*]N(C)c1ncc(F)cn1	CN(c1ncc(F)cn1)C1CCc2c(CC(=O)O)c3ccc(Cl)cc3n2C1	-33.05	0.83	1.0		8.0		

# Ligand annotations

uniprot_ID	MotifLead_ligand_ID	ligand_SMILES	pKi	pIC50	pKd	pEC50	BindingDB_ligand_IDs	PubChem_CID	PubChem_SID	ChEMBL_ID	DrugBank_ID	ZINC_ID
Q9Y5Y4	Q9Y5Y4.MLD104703	CN(c1ncc(F)cn1)C1CCc2c(CC(=O)O)c3ccc(Cl)cc3n2C1		8.0			318337	66571710.0	312697205.0			

# BindingDB-derived annotations

BindingDB_ligand_ID	pKi	pIC50	pKd	pEC50	pH	Temp(C)
318337		10.0			7	25.00

# Target metadata

uniprot_ID	BindingDB_target_ID	target_name	organism	protein_function	sequence
Q9Y5Y4	BDBpoly_5608	Prostaglandin D2 receptor 2	Homo sapiens (Human)	G-protein coupled receptor,Receptor,Transducer	MSANATLKPLCPILEQMSRLQSH...

Supplementary Table S5. Description of metadata columns in MotifLeadDB. This table describes all columns used across the metadata and tables CSV files in MotifLeadDB, summarizing structural, chemical, and energetic information for receptor–ligand complexes.

Column name	Description	Example value
target_name	Common name of the protein target.	Prostaglandin D2 receptor 2
uniprot_ID	UniProt accession ID of the target protein.	Q9Y5Y4
BindingDB_target_ID	Target identifier derived from BindingDB.	BDBpoly_5608
organism	Source organism of the target protein.	Homo sapiens (Human)
protein_function	Functional annotation of the target protein.	G-protein coupled receptor,Receptor,Transducer
sequence	Amino acid sequence of the target protein (UniProt reference sequence).	MSANATLKPLCPILEQMSRLQSH…
receptor_id	Identifier of the template receptor complex used for modeling (UniProt ID + PDB ID + ligand ID).	Q9Y5Y4_6D27_FT4
scaffold_id	Identifier of the scaffold group used for modeling (receptor_id + scaffold index).	Q9Y5Y4_6D27_FT4_9
model_id	Identifier of the modeled protein–ligand complex (scaffold_id + model index).	Q9Y5Y4_6D27_FT4_9_1
group_SMILES	Generic SMILES representation of the scaffold (attachment points denoted as [*]).	[*]C1CCc2c(CC(=O)O)c3ccc(Cl)cc3n2C1
substituent_SMILES	SMILES representation of the substituent attached to the scaffold.	[*]N(C)c1ncc(F)cn1
ligand_SMILES	Canonical SMILES of the ligand.	CN(c1ncc(F)cn1)C1CCc2c(CC(=O)O)c3ccc(Cl)cc3n2C1
pKi, pIC50, pKd	Experimental activity values collected from BindingDB (negative log scale). When multiple measurements were available for the same ligand, the median value was used.	nan, 8.0, nan
dH	Docking enthalpy term (ΔH).	-33.05
GDT-HA	Score measuring the preservation of the template scaffold geometry in the modeled ligand pose.	0.83
pharmacophore_score	Pharmacophore matching score.	1.0
confidence_level	Final confidence level of the model. (Level 1–3).	1
MotifLead_ligand_ID	Unique MotifLeadDB ligand identifier assigned to each distinct ligand SMILES (UniProt ID + MotifLeadDB ligand index).	Q9Y5Y4.MLD104703
BindingDB_ligand_IDs	List of BindingDB ligand identifiers associated with the same ligand SMILES, whose experimental values were aggregated and used in MotifLeadDB.	318337
PubChem_CID	PubChem Compound ID of the ligand.	66571710.0
PubChem_SID	PubChem Substance ID of the ligand.	312697205.0
ChEMBL_ID	ChEMBL identifier of the ligand, when available.	
DrugBank_ID	DrugBank identifier of the ligand, when available.	
ZINC_ID	ZINC database identifier of the ligand, when available.	
BindingDB_ligand_ID	Ligand identifier from BindingDB.	318337
pH	Experimental pH condition reported for the activity measurement.	7
Temp(C)	Experimental temperature (°C) reported for the activity measurement.	25.00
available_activity_type	activity-type	pIC50

Supplementary Figure S1. Trade-off between minimum ligand-count threshold and retained targets.

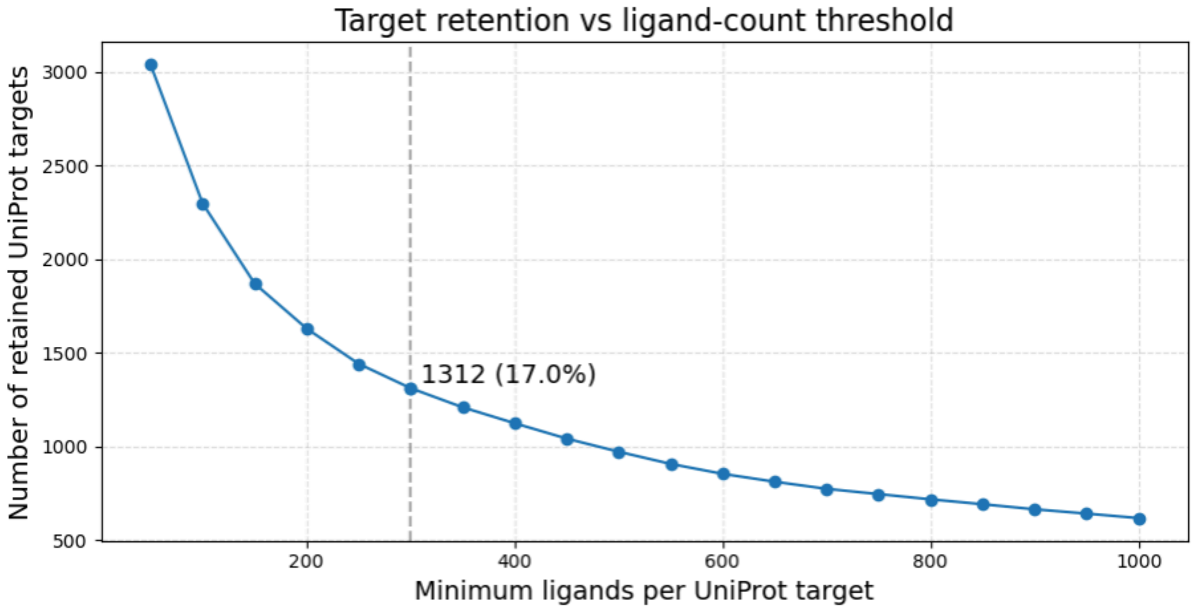

Supplementary Figure S2. Distribution of heavy-atom counts for FDA-approved small-molecule drugs. The dashed line indicates the cutoff used in this study (≤35 heavy atoms). This threshold retains 82.2% of FDA-approved small molecules (cumulative curve), thereby focusing downstream analyses on drug-like chemical space and reducing outliers from very large ligands.

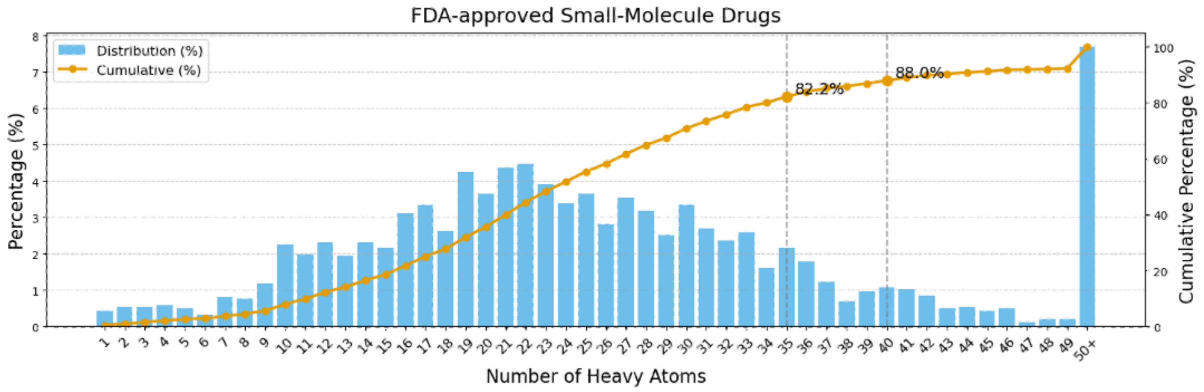

Supplementary Figure S3. Examples of scaffold annotation using BRICS and MCS. (a) BRICS-based scaffold definition. A ligand is fragmented at BRICS-defined bond positions (arrows 1–3). For each cleavage, the larger fragment is assigned as the scaffold, and the smaller fragment is assigned as the substituent. (b) MCS-based scaffold definition. The maximum common substructure is identified between the template ligand and a chemically similar ligand within the same similarity cluster. The shared substructure (highlighted in red) is used as the scaffold, and the ligand-specific remaining parts (labeled 1* and 2*) are treated as substituents.

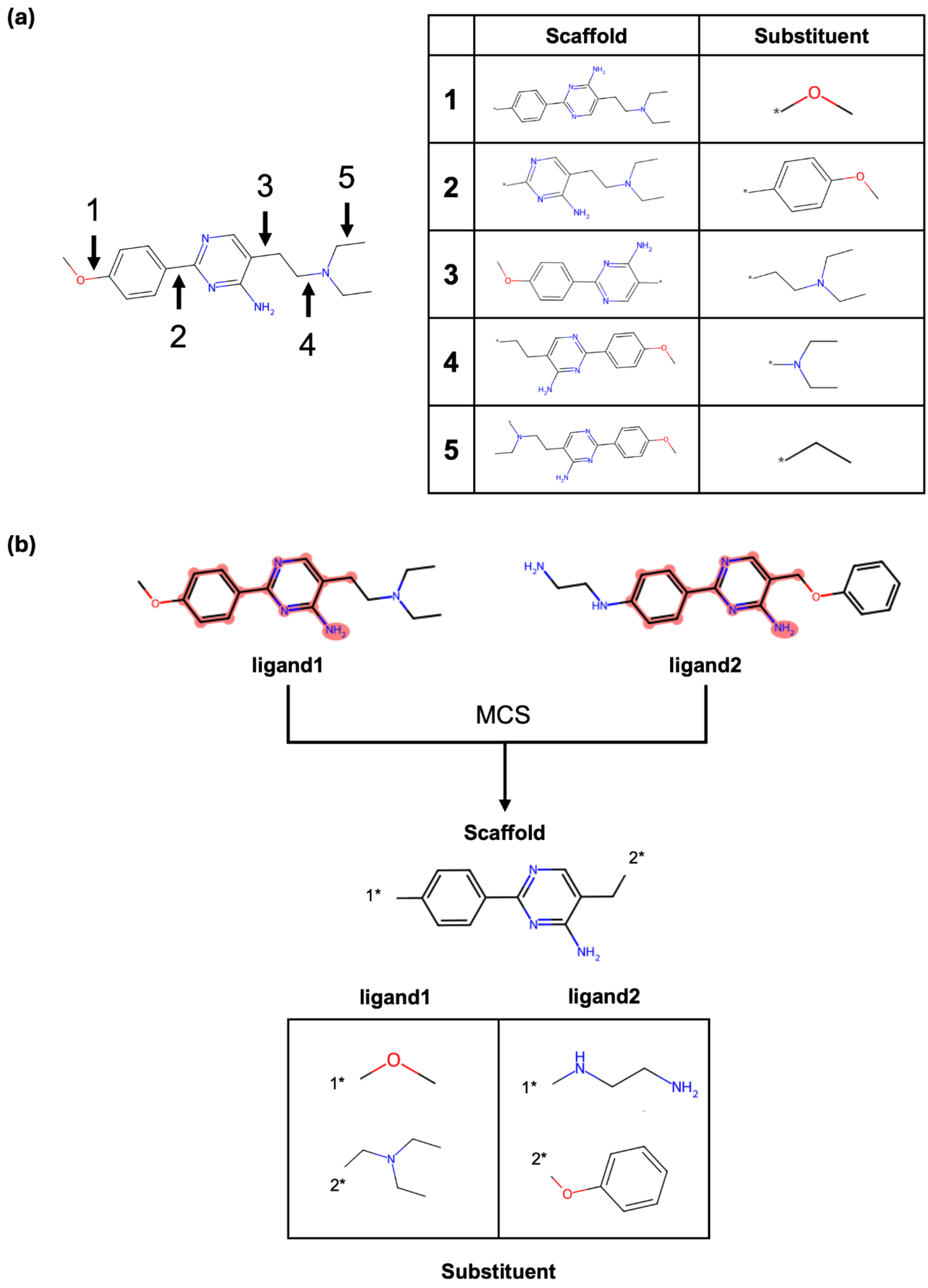

Supplementary Figure S4. Scaffold group formation and supergroup unification. (a, b) Multiple ligands sharing a common chemical scaffold (highlighted) are decomposed via BRICS or MCS analysis to extract the shared scaffold. Ligands sharing the same scaffold are assembled into a scaffold group; two example groups (α and β) are illustrated. (c) Two scaffold groups sharing one or more ligands are merged into a single supergroup. In this example, ligand A1 of group α is structurally identical to ligand B1 of group β, serving as the bridging shared ligand (red box) that links the two groups. The merged supergroup (α ∪ β) contains five distinct ligands. A single experimental complex is then assigned as the template for all members of the supergroup.

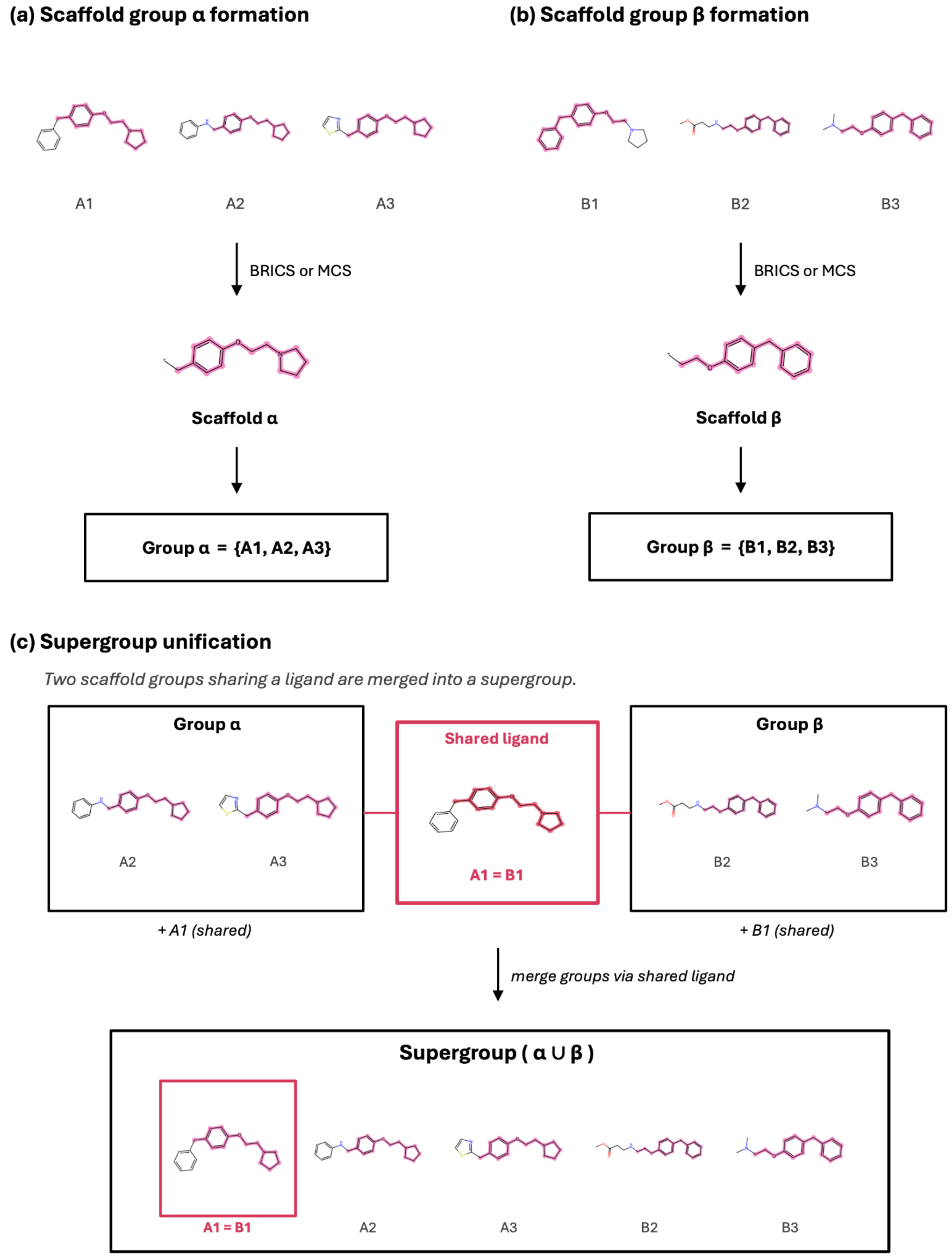

Supplementary Figure S5. Tanimoto similarity statistics in template assignment. (a) Distribution of scaffold–template Tanimoto similarity values, counted per unique ligand–template pair, considered during intermediate template assignment. (b) Cumulative fraction of unique ligand–template pairs retained as a function of the scaffold–template Tanimoto cutoff. The cumulative fraction decreases from ~25% at a cutoff of 0.5 to ~14% at 0.6, supporting the use of 0.5 as a practical compromise between similarity stringency and scaffold-group coverage. (c) Distribution of mean scaffold–template Tanimoto similarity per supergroup, computed for supergroups in the final dataset (n = 1,877). Most supergroups show average Tanimoto ≥ 0.6 (mean = 0.66, median = 0.65).

(a)

(b)

(c)

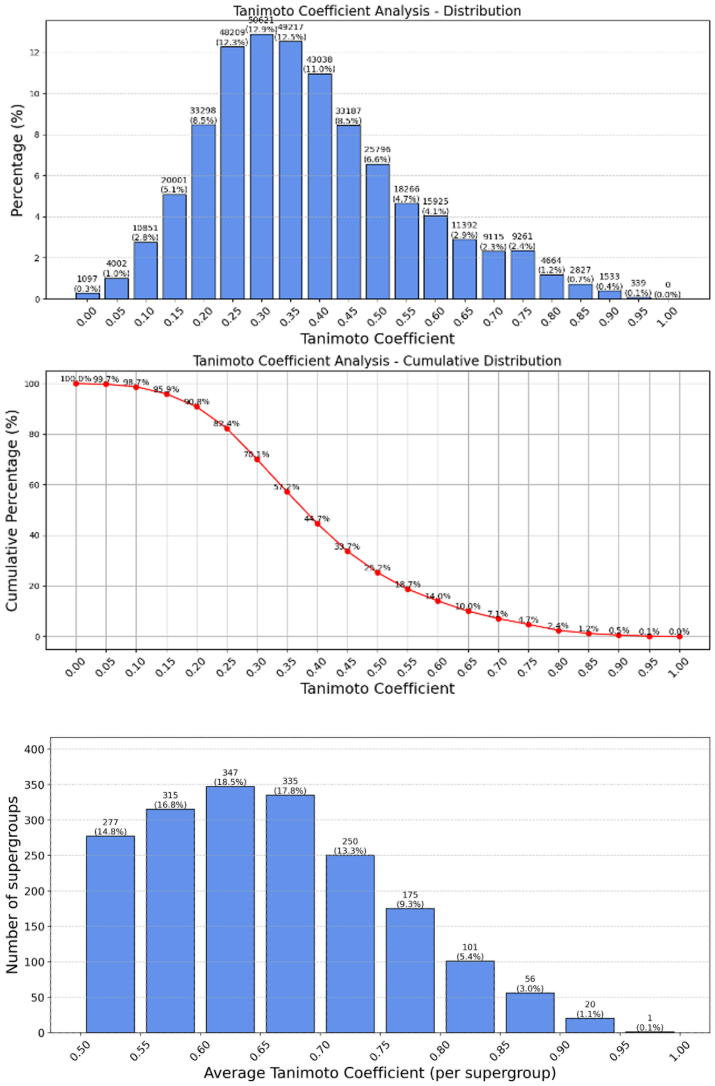

Supplementary Figure S6. Distribution and structural consistency of ligands assigned to multiple scaffold groups. (a) Distribution of the number of scaffold groups associated with each ligand, showing that the majority of ligands were linked to one to three scaffold definitions due to independent scaffold grouping. (b) Distribution of mean GDT-HA_{lig} values among ligands appearing in multiple scaffold groups, showing moderate overall structural consistency (mean = 0.601). Although perfect overlap was not always achieved, most multi-scaffold ligands retained comparable binding geometries.

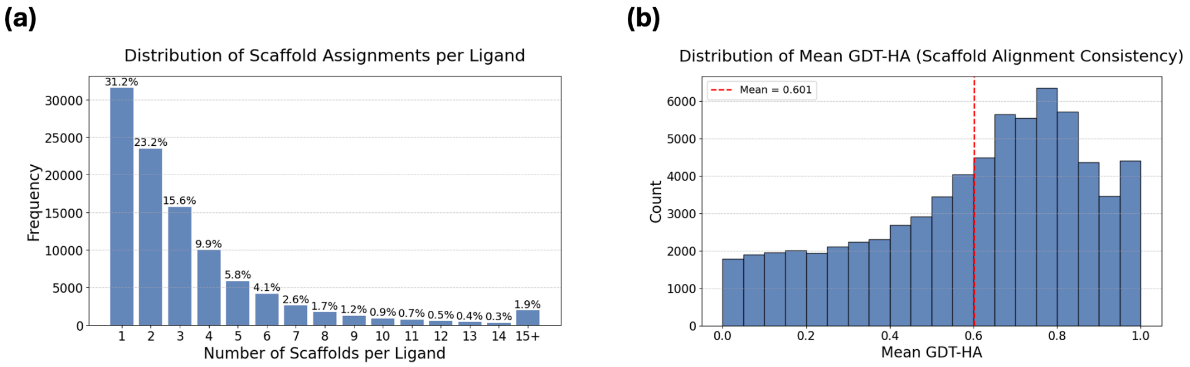

Supplementary Figure S7. Supplementary targets added to expand the Diverse set. The added branch contributed 24 target proteins, 1,754 ligands, and 6,009 modeled entries. Left, target-class distribution of the added targets. Right, summary counts for the supplementary branch.

(a)

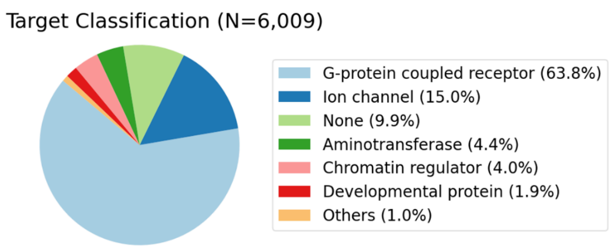

(b)

	**Added Diverse set branch**
**Num. target proteins**	24
**Num. Ligands**	1,754
**Grouped by Ligand scaffold**	1,089
**Complex structures p rovided**	6,009

Supplementary Figure S8. Activity-type composition and provenance-aware activity analysis in the Core set. (a) Activity-type composition of retained annotations in the Core set. (b) Provenance consistency across all same-type scaffold groups containing at least two ligands, assessed using PMID-, DOI-, and patent-based source signatures. (c) Provenance of large-range scaffold groups, assessed using the range-defining max–min ligand pairs for groups with ΔpActivity ≥ 2.0. “All same source” indicates that all ligands in the group shared at least one common source signature; “Mixed source” indicates that provenance was available but not shared across all ligands; “Not assessable” indicates that source metadata were missing for one or more ligands.

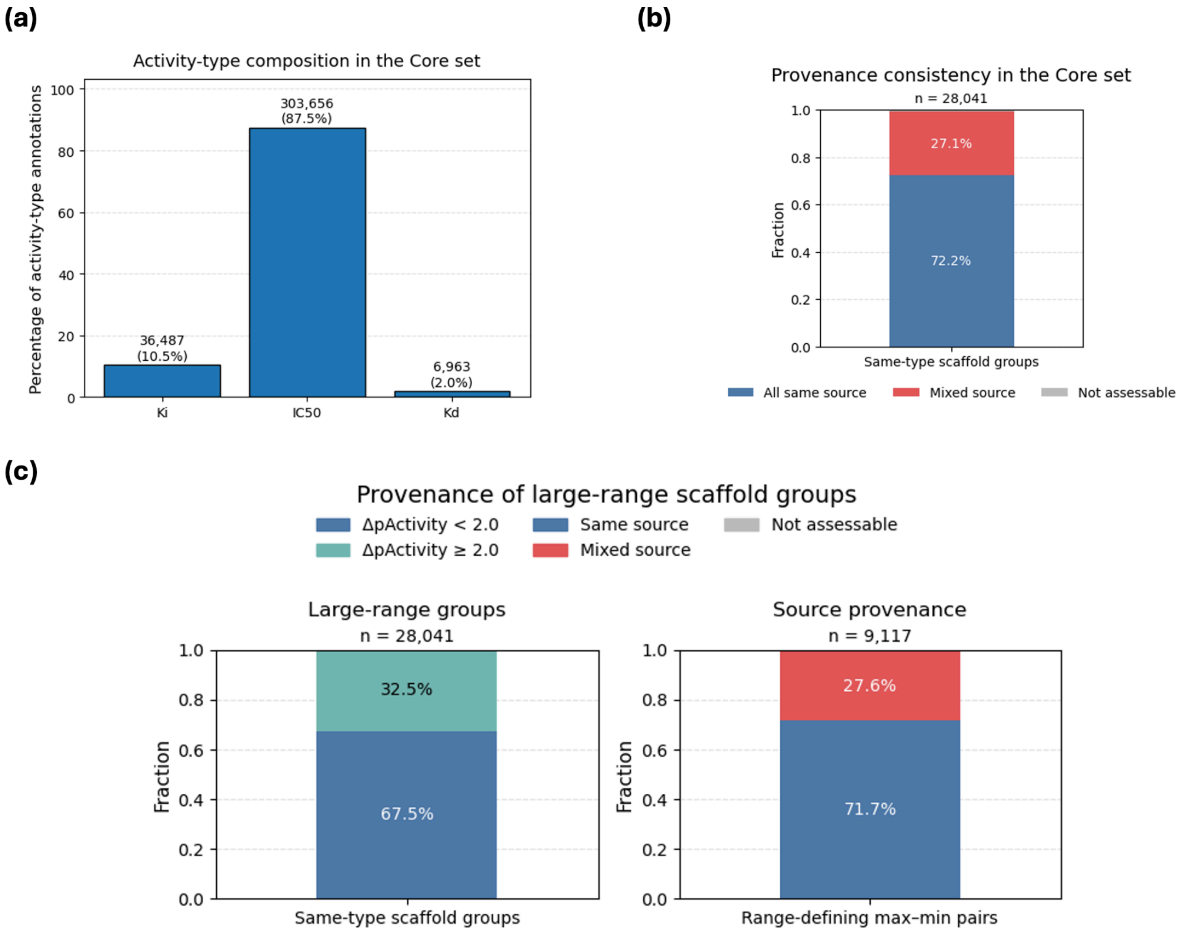

Supplementary Figure S9. **Scaffold statistics.** (a) Distribution of unique scaffold sizes (number of heavy atoms). (b) Cumulative ligand coverage curve by scaffold rank. (c) Representative examples of the top 20 most frequent scaffolds.

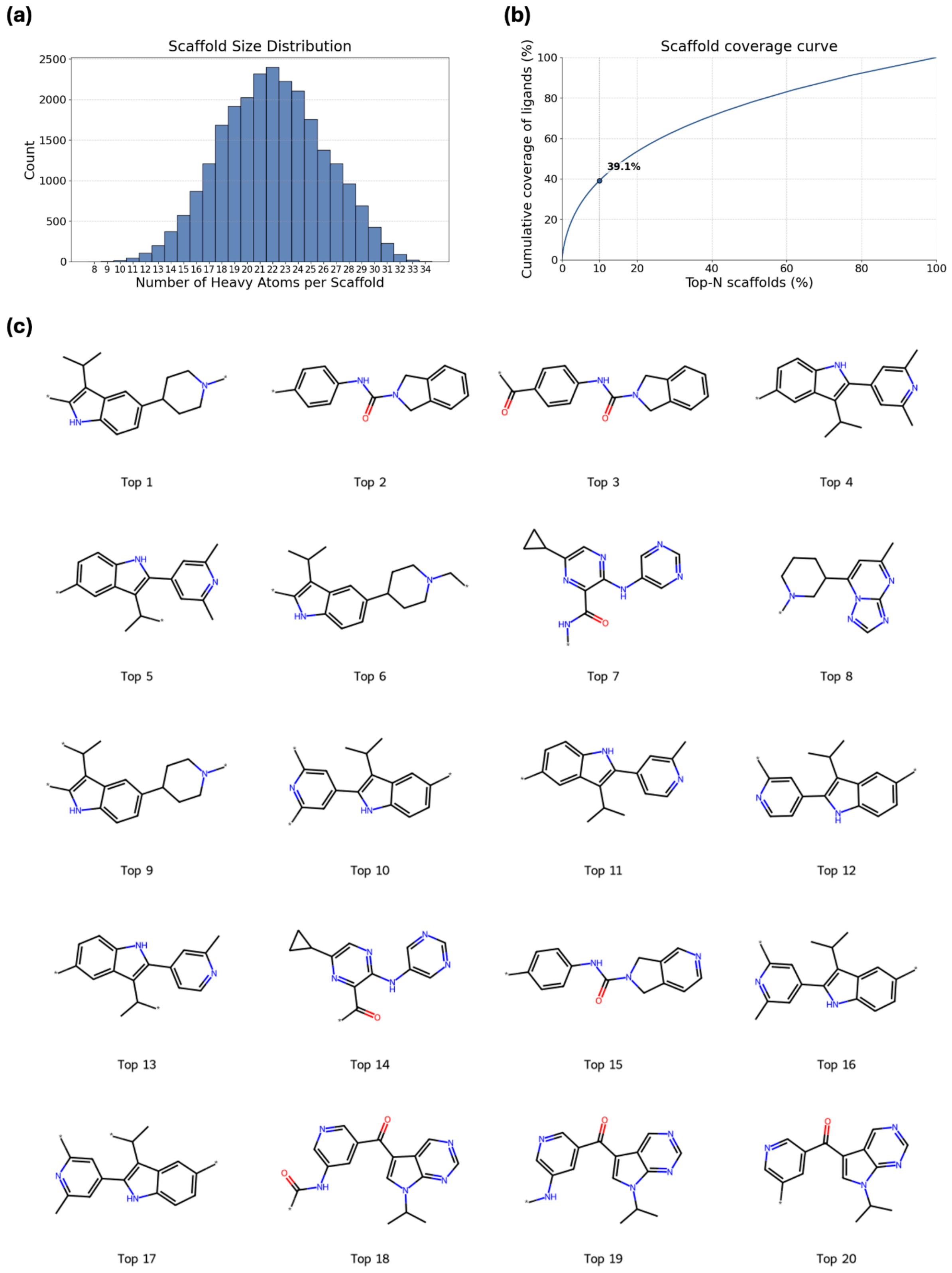

Supplementary Figure S10. Substituent statistics. (a) Distribution of unique substituents sizes (number of heavy atoms). (b) Number of substituents per ligand. (c) Representative examples of the top 20 most frequent substituents.

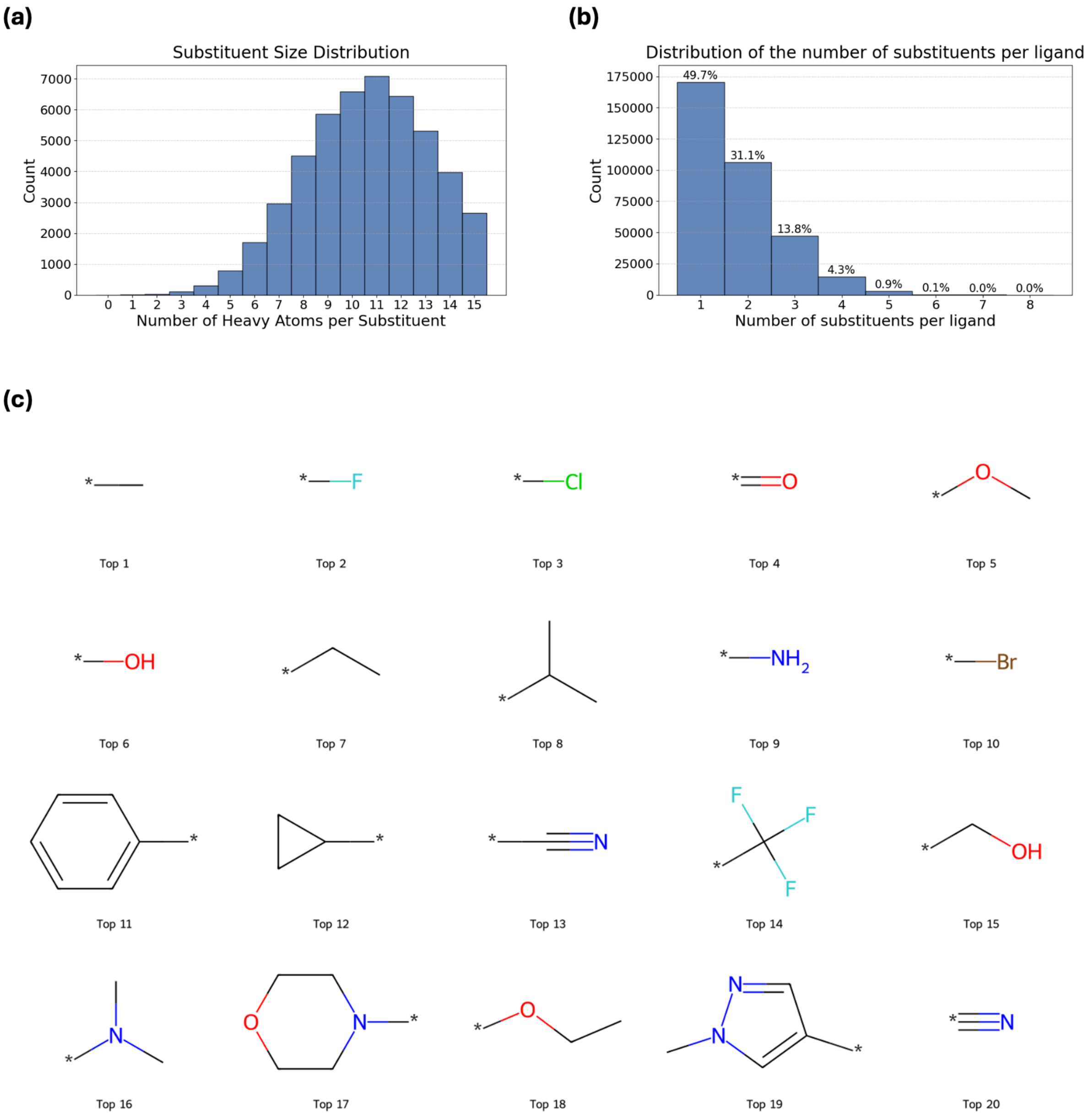

Supplementary Figure S11. Confidence assessment statistics. (a) Distribution of ligand GDT-HA scores used for confidence classification. Over 60% of ligands achieved GDT-HA ≥ 0.7, indicating strong structural agreement with template scaffolds. (b) Distribution of pharmacophore conservation scores. Most ligands retained ≥ 0.8 feature similarity, consistent with high chemical correspondence between modeled and template poses.

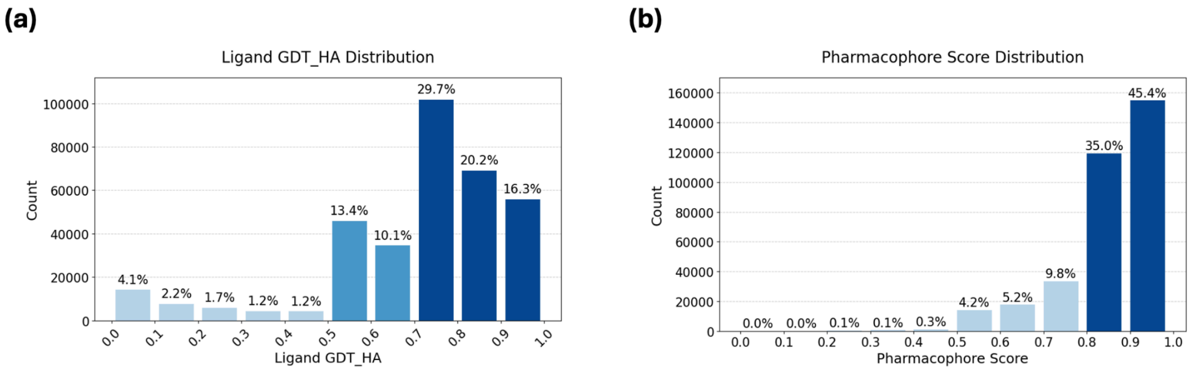

Supplementary Figure S12. Additional analyses of the crystal-validated Core benchmark subset. (a) Distribution of ligand RMSD across confidence levels. High-confidence (Level 1) models generally exhibited lower RMSD values, whereas lower-confidence models showed broader and higher RMSD distributions. (b) Comparison of RMSD between scaffold and substituent atoms for ligands with full RMSD > 2 Å. Deviations were primarily localized in peripheral substituent regions, while the core scaffold geometry remained largely preserved. (c) Representativeness of the crystal-validated Core benchmark subset relative to the parent Core set, shown by the target-class distribution (left) and confidence-tier distribution (right). The benchmark subset broadly preserved the dominant kinase/enzyme composition of the Core set, but underrepresented GPCR and receptor classes and was enriched for higher-confidence entries, particularly Level 1 models.

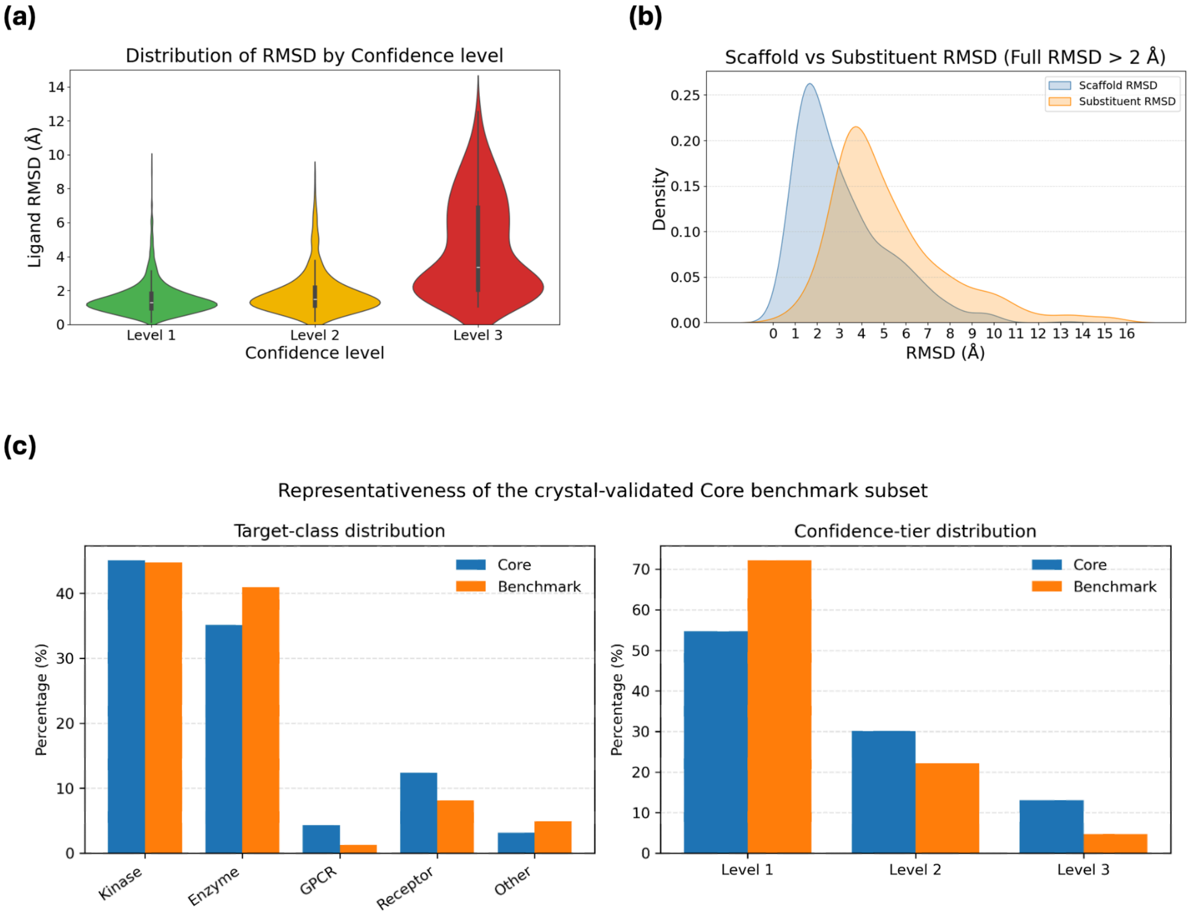

Supplementary Figure S13. Statistical summary of side-chain remodeling across the dataset. (a) Distribution of the number of residues per pocket exhibiting significant side-chain rotations ($\Delta\chi \geq 60°$). (b) Residue-type frequency of rotameric changes. (c) Relationship between the number of side-chain rotations and the change in clashing residues.

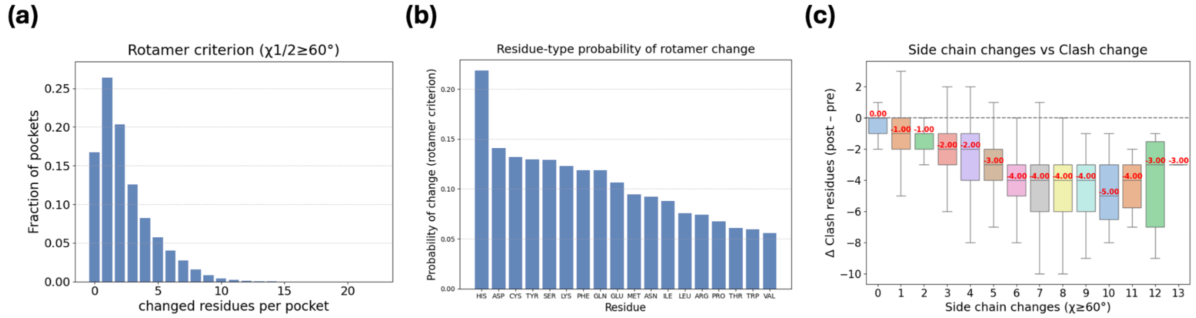

Supplementary Figure S14. Changes in noncovalent interactions upon side-chain remodeling. Distribution of the changes in major noncovalent interactions (Δn = post − pre), including hydrogen bonds, salt bridges, hydrophobic contacts, and π–π stacking interactions.

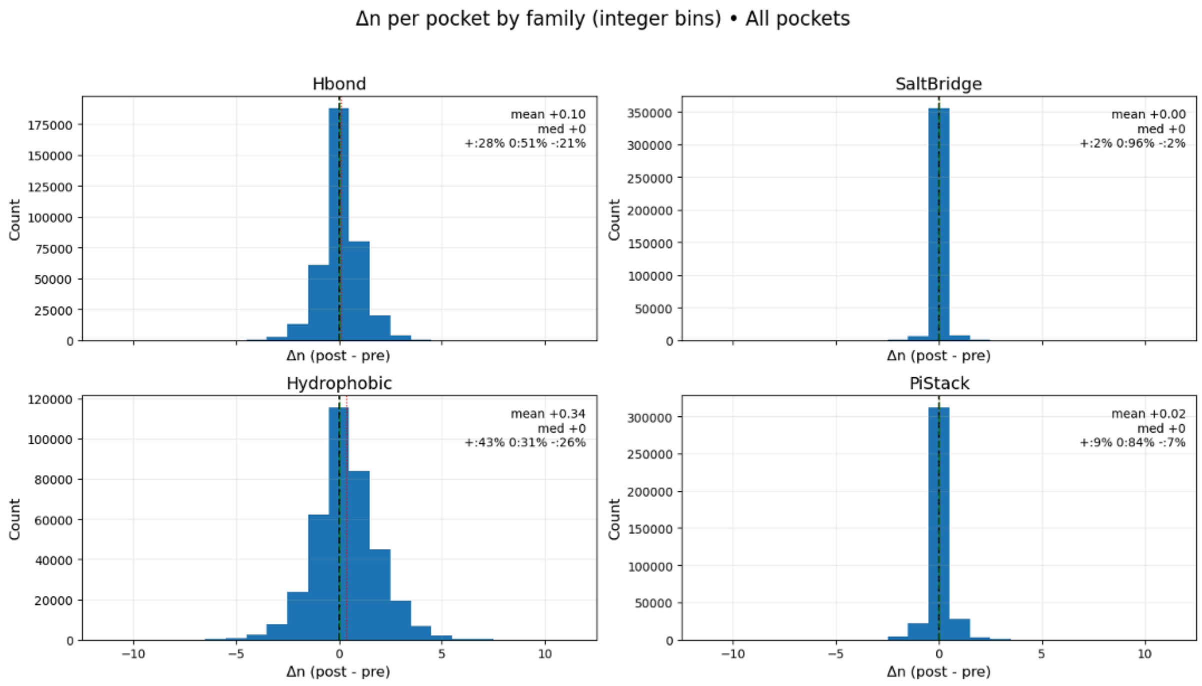

Supplementary Figure S15. Distribution of substituent heavy-atom counts before applying the 15-heavy-atom cutoff. The dashed vertical line indicates the cutoff used during scaffold-group filtering. Only 2.62% of substituents exceeded this threshold.

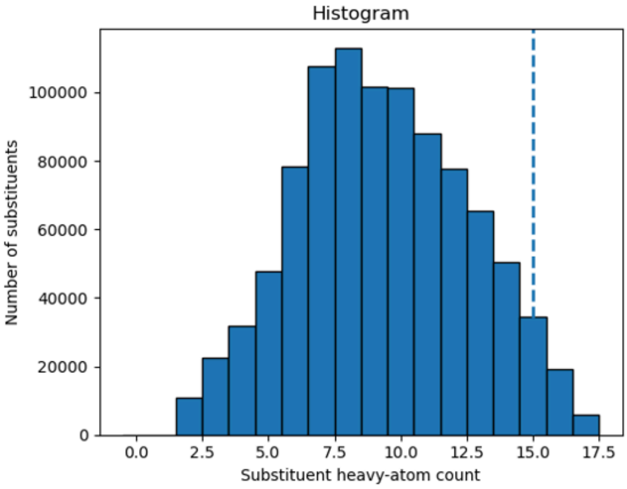

Supplement: Supplementary file 1 [file ci6c00128_si_001.pdf]
